# Unveiling the early life core microbiome of the sea cucumber *Apostichopus japonicus* and the unexpected abundance of the growth-promoting *Sulfitobacter*

**DOI:** 10.1186/s42523-023-00276-2

**Published:** 2023-10-24

**Authors:** Juanwen Yu, Chunqi Jiang, Ryota Yamano, Shotaro Koike, Yuichi Sakai, Sayaka Mino, Tomoo Sawabe

**Affiliations:** 1 Laboratory of Microbiology, Faculty of Fisheries Sciences, Hokkaido University, Hakodate, Japan; 2 Hakodate Fisheries Research, Hokkaido Research Organization, Local Independent Administrative Agency, Hakodate, Japan; 3 Atmosphere and Ocean Research Institute, The University of Tokyo, Chiba, Japan

**Keywords:** Core microbiome, *Apostichopus japonicus*, Larval ontogenesis, Host-microbe interaction, Meta-pangenomics

## Abstract

**Background:**

Microbiome in early life has long-term effects on the host’s immunological and physiological development and its disturbance is known to trigger various diseases in host Deuterostome animals. The sea cucumber *Apostichopus japonicus* is one of the most valuable marine Deuterostome invertebrates in Asia and a model animal in regeneration studies. To understand factors that impact on host development and holobiont maintenance, host-microbiome association has been actively studied in the last decade. However, we currently lack knowledge of early life core microbiome during its ontogenesis and how it benefits the host’s growth.

**Results:**

We analyzed the microbial community in 28 sea cucumber samples from a laboratory breeding system, designed to replicate aquaculture environments, across six developmental stages (fertilized eggs to the juvenile stage) over a three years-period to examine the microbiomes’ dynamics and stability. Microbiome shifts occurred during sea cucumber larval ontogenesis in every case. Application of the most sophisticated core microbiome extraction methodology, a hybrid approach with abundance-occupancy core microbiome analyses (top 75% of total reads and > 70% occupation) and core index calculation, first revealed early life core microbiome consisted of *Alteromonadaceae* and *Rhodobacteraceae*, as well as a stage core microbiome consisting of pioneer core microbe *Pseudoalteromonadaceae* in *A. japonicus*, suggesting a stepwise establishment of microbiome related to ontogenesis and feeding behavior in *A. japonicus*. More interestingly, four ASVs affiliated to *Alteromonadaceae* and *Rhodobacteraceae* were extracted as early life core microbiome. One of the ASV (ASV0007) was affiliated to the *Sulfitobactor* strain BL28 (*Rhodobacteraceae*), isolated from blastula larvae in the 2019 raring batch. Unexpectedly, a bioassay revealed the BL28 strain retains a host growth-promoting ability. Further meta-pangenomics approach revealed the BL28 genome reads were abundant in the metagenomic sequence pool, in particular, in that of post-gut development in early life stages of *A. japonicus*.

**Conclusion:**

Repeated rearing efforts of *A. japonicus* using laboratory aquaculture replicating aquaculture environments and hybrid core microbiome extraction approach first revealed particular ASVs affiliated to *Alteromonadaceae* and *Rhodobacteraceae* as the *A. japonicus* early life core microbiome. Further bioassay revealed the growth promoting ability to the host sea cucumber in one of the core microbes, the *Sulfitobactor* strain BL28 identified as ASV0007. Genome reads of the BL28 were abundant in post-gut development of *A. japonicus*, which makes us consider effective probiotic uses of those core microbiome for sea cucumber resource production and conservation. The study also emphasizes the importance of the core microbiome in influencing early life stages in marine invertebrates. Understanding these dynamics could offer pathways to improve growth, immunity, and disease resistance in marine invertebrates.

**Supplementary Information:**

The online version contains supplementary material available at 10.1186/s42523-023-00276-2.

## Background

Early life microbiome has been intensively studied in recent decades since the establishment of gut microbiota has long-term effects on immunological and physiological development [1]. Diseases including obesity, diabetes, inflammatory bowel disease, cancer, allergies, asthma, and neurological diseases are all linked with gut homeostasis during early life [2–4]. The dynamic changes in infants’ microbiome during the first year after birth are influenced by delivery mode and feeding, indicating early life gut microbiome gradually develops and matures to adult-like microbiomes with core bacteria *Firmicutes* and *Bacteroidetes*, followed by *Actinobacteria* and *Proteobacteria* [5]. Core microbiome is defined as a group of microbes that are shared within host populations, which are particularly essential to the host biological functions such as health maintenance, disease resistance and nutrient intake [6, 7]. With the development of studies in host-associated microbiome, the definition of core microbiome has evolved to a more complicated and advanced explanation: common core, temporary core, ecological core, functional core and host-adapted core microbiome [8]. The established core microbiome is used as a reference to identify diseased organisms and an indicator of good health [9, 10]. In the marine environment, research has identified a core microbiome within aquatic organisms that is influenced by geographic location, climate change, and seasonal changes [11–14]. This core microbiome plays a crucial role in maintaining the health of the host organism and facilitating adaptations to its environment [11–14]. However, our knowledge of core microbiome during host ontogenesis and microbial establishment is very limited.

Sea cucumber *A. japonicus* (Selenka, 1867), belonging to Echinodermata, Holothuroidea, is one of the most valuable marine invertebrates in Asia for its biological factors, economic benefits and phylogenetic position [15]. Since the exclusive application of *A. japonicus* in the food, cosmetic and medical industries, *A. japonicus* has been placed on the red list of endangered species due to the over-exploitation of wild sea cucumber [16]. Increasing studies investigating factors impact on sea cucumber’s aquaculture include animal density, culture condition, diets, genetic factors and host-associated microbiome, in which the study on gut microbiome of sea cucumber give us new insights into how microbes contribute to its growth, immunity and disease [17–20]. For example, the probiotic dietary supplements of *Pseudoalteromonas elyakovii* HS1, *Shewanella japonica* HS7, and *Vibrio tasmaniensis* HS10 can improve growth, immune responses and survival rate of sea cucumber [21]. *Bacillus subtilis* T13, as a dietary probiotic, is able to decrease the infection of *Vibrio splendidus*, which highly improved the resistance to *V. splendidus* related diseases such as skin ulceration syndrome [16, 22]. Comparison of individual sea cucumber with different growth gap revealed *Rhodobacteraceae* related with polyhydroxybutyrate (PHB) metabolism gene in *A. japonicus* are significantly abundant in larger individuals, suggesting the *Rhodobacteraceae* may benefit to host growth [23]. In addition, studies on the re-assembly of the gut microbiome after evisceration emphasize host-associated microbiome plays an important role in gut regeneration and functional recovery [24, 25]. Previous study on the establishment of early life microbiome during larval development in sea cucumber revealed dynamic changes of microbiota through seven developmental stages: fertilized egg (FE) and blastula (BL), gastrula (GL), early auricularia (EA), late auricularia (LA), pentactula (PT) and juvenile (JN), which is probably related to larval ontogenesis [26]. However, due to the limitation of sample size in laboratory settings and microbial functional analysis, the early life core microbiome and their related functions in host biology are still poorly understood.

Meta-pangenomics is a novel approach combining pangenomes and metagenomes to investigate bacterial genomes recruited reads in their environmental metagenomes, providing broader perspectives of understanding the roles of its distribution and biogeographical patterns through the microbial population [27, 28]. Meta-pangenomics have been applied to investigate the human microbiome, revealing the distribution of identical genes in pangenomes across populations in different locations and ages [28, 29]. The adaptation of microbial populations in extreme environment from deep sea microbiome also has been investigated by meta-pangenomics [30, 31]. However, there has been no study applied meta-pangenomics on the distribution of host-associated microbes in host microbiome during early life development.

Here, we performed metagenomic analysis to assess the dynamics of microbiome during the larval ontogenesis in sea cucumber and to detect the core microbiome in early life stages. We also applied meta-pangenomics to underscore how specific bacteria and genes are distributed in different developmental stages, and discovery probiotic bacteria promote host growth from early stages. Our results demonstrate the detection of early life core microbiome and stage core microbiome in *A. japonicus* using an abundance-occupancy core microbiome analysis, which is significantly related to ontogenesis and feeding behavior, suggesting a stepwise established microbiome associated with the *A. japonicus* host.

## Results

### The dynamics of microbial diversity and composition during larval development

A total of 2,337,684 Meta16S sequence reads were obtained from 28 sea cucumber and 26 seawater samples collected from six stages of FE, GL, EA, LA, PT and JN, in 2019, 2020 and 2021. Reads passed quality control and removed eukaryotic reads (e.g., mitochondria and chloroplast) were used for microbial diversity analyses and taxonomic assignments; 465,273 and 599,246 qualified reads were generated from sea cucumber samples and seawater samples, respectively.

**Fig. 1:**
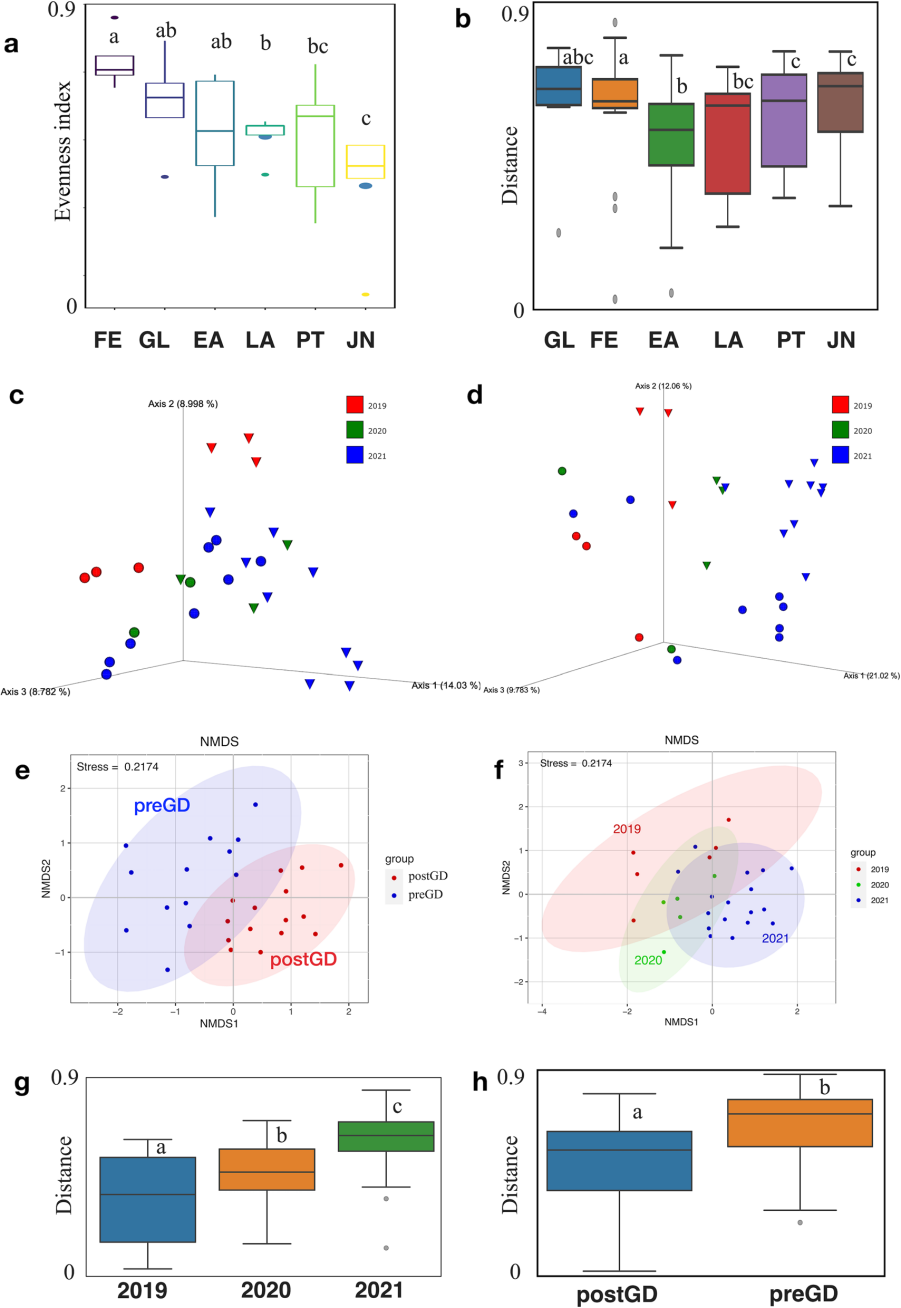
Alpha and beta diversity among sea cucumber samples. **a** Box-plot based on evenness index (Kruskal-Wallis, *p* < 0.05). **b** Unweighted UniFrac distance plot between sea cucumber larvae at different developmental stage (PERMANOVA, *p* < 0.05, *q* < 0.05). FE: Fertilized Egg; GL: Gastrula; EA: Early Auricularia; LA: Late Auricularia; PT: Pentactula; JN: Juvenile. **c** PCoA plot based on Bray–Curtis distance and **d** PCoA plot based on Unweighted UniFrac distance. Different colors represent different years; closed circles represent larvae samples before gut development, triangles represent larvae samples post gut development. **e** NMDS (non-metric multidimensional scaling) ordination based on Bray–Curtis distance showing the differences of microbiome both pre and post gut development. preGD: pre gut development; postGD: post gut development. **f** NMDS ordination based on Bray–Curtis distance showing the differences of microbiome between years. **g** Unweighted UniFrac distance plot between different years ((PERMANOVA, *p* = 0.001, *q* < 0.05) and **h** Unweighted Unifrac distance plot between sea cucumber larvae before and post gut development (PERMANOVA, *p* = 0.001, *q* < 0.05)

In order to understand the fluctuations of microbial communities during sea cucumber’s larval development, a microbial diversity analysis was performed. Although alpha diversity based on evenness significantly changed within developmental stages, the initial stages before gut development (FE to EA) are generally higher than later stages, especially increasing at the FE and GL stages (Fig. 1a). Unweighted and weighted UniFrac distance analysis revealed the microbial community changed along with the larval developmental stages (Fig. 1b and Additional file 2: Fig. S6). In particular, the microbiota is significantly different between the EA and LA stages (*p* < 0.01), indicating microbiota changes before and after the gut developed at that point (Fig. 1c–e and h). Additionally, it is worth noting that the microbiota compositions of larvae from different years fluctuated, as the results of beta diversity analyses (Fig. 1f and g, and Additional file 2: Fig. S6). Following the gut development, we observed a decrease in alpha diversity alongside an increase in beta diversity of the microbiotas (Fig. 1a and b).

### The microbial composition of early life sea cucumber at different stages

**Fig. 2:**
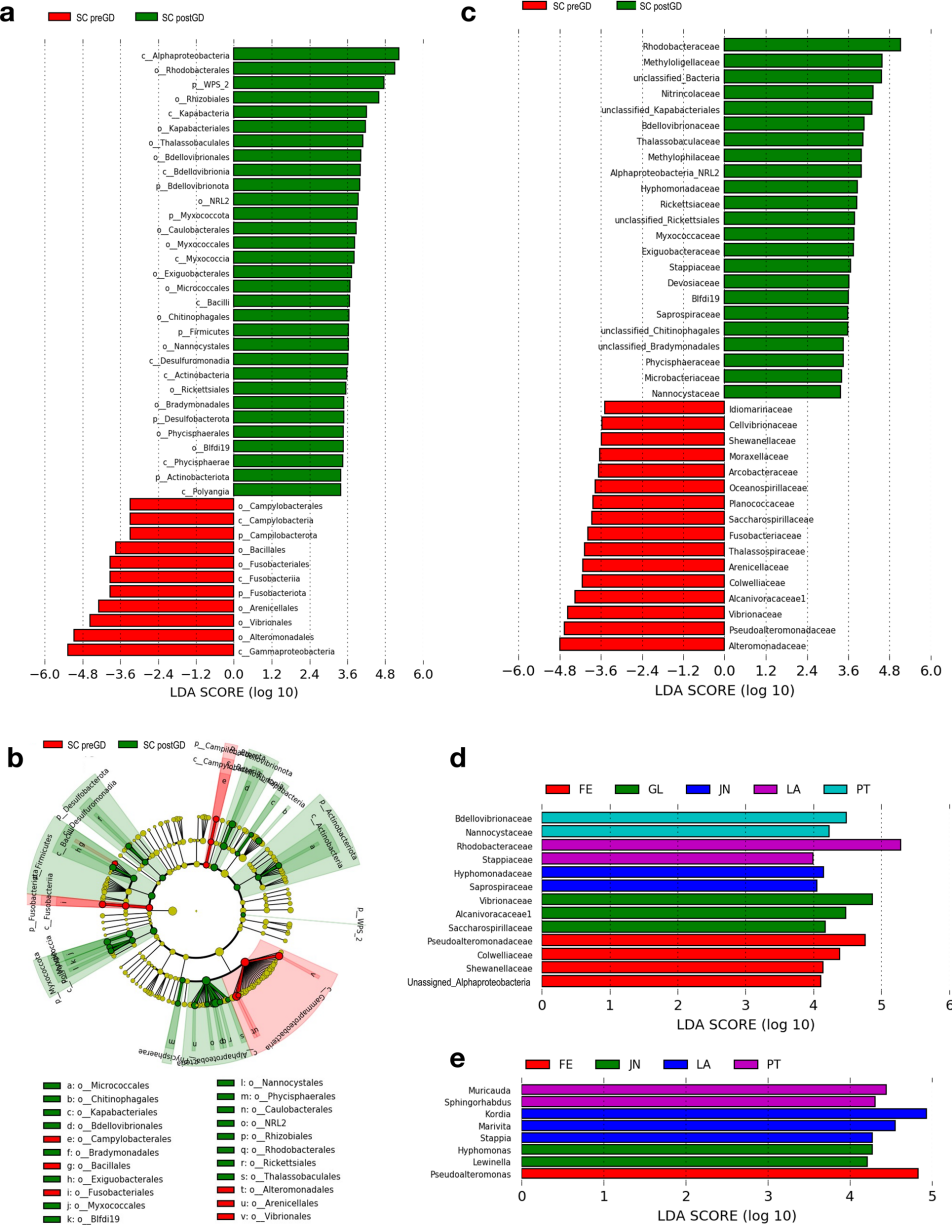
The linear discriminant analysis effect size (LEfSe) analysis of microbial abundance among sea cucumber larvae samples at different developmental stage. **a** Taxa until order level with significant differences pre- and post-gut development were detected by LEfSe analysis with a LDA threshold score of 3.5 and a p-value of 0.05. **b** The cladogram of detected prokaryotic taxa for microbial community pre- and post-gut development. **c** Only family level taxa with significant differences pre- and post-gut development were detected by LEfSe analysis with a LDA threshold score of 3.5 and a p-value of 0.05. **d** Only family level taxa with significant differences at each developmental stage were detected by LEfSe analysis with a LDA threshold score of 3.5 and a p-value of 0.05. **e** Only genus level taxa with significant differences at each developmental stage were detected by LEfSe analysis with a LDA threshold score of 3.5 and a p-value of 0.05

A total of 2,237 ASVs from sea cucumber samples was obtained and further assigned to 93 orders, 152 families and 332 genera using a similarity threshold of 99% sequence identity. On order-level taxonomic rank, *Alteromonadales* (31.2 ± 23.3%) ([mean ± SD]) and *Rhodobacteriales* (22.9 ± 17.9%) are the most dominant bacterial taxa in early life stages, followed with *Oceanospirillales* (12.8 ± 17.1%) and *Flavobacteriales* (7.4 ± 8.3%) (Fig. 2a and b, and Additional file 1: Table S1.

**Fig. 3:**
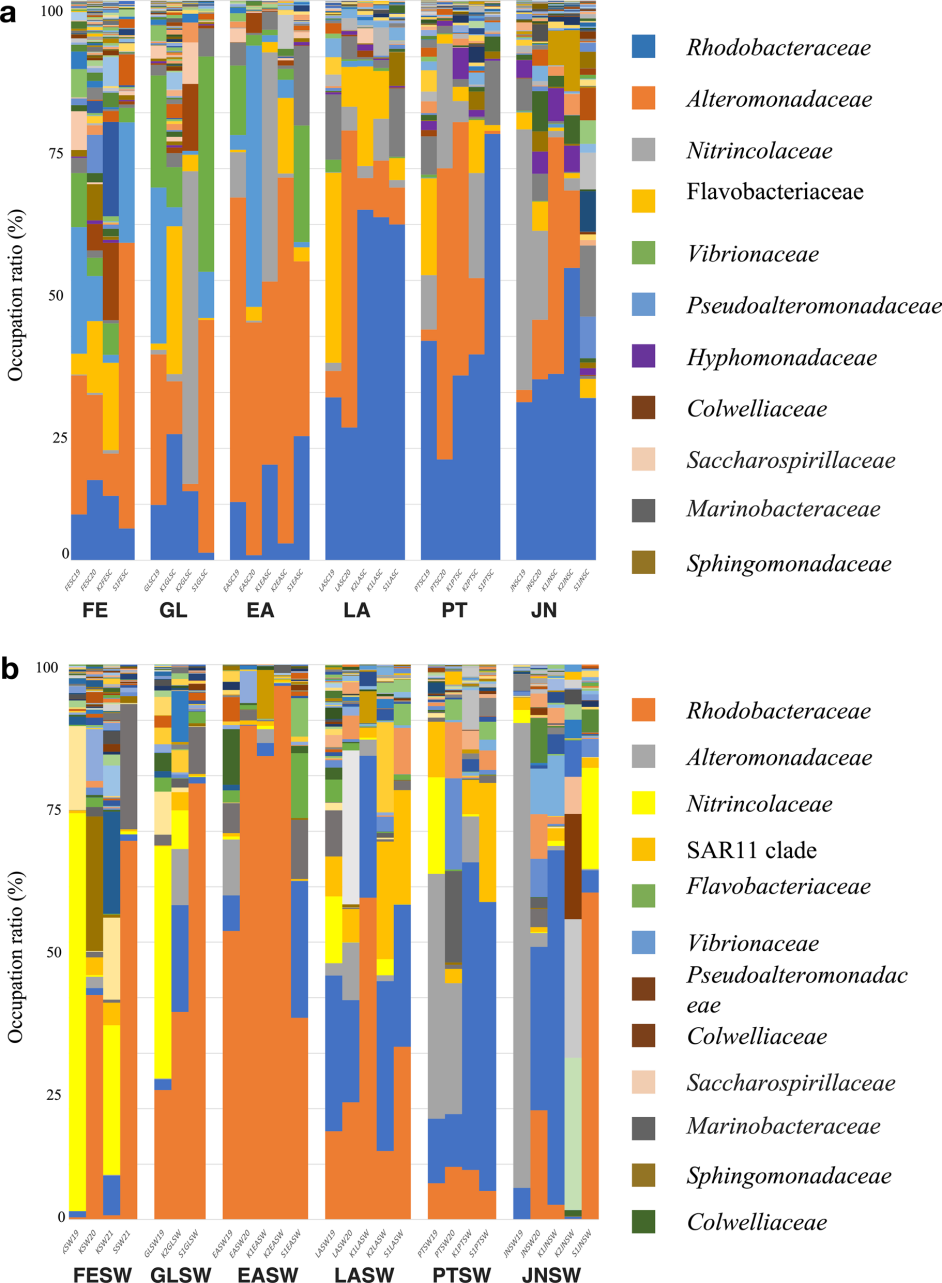
Family-level taxonomic distribution among sea cucumber and seawater samples. **a** Family-level taxonomic distribution among sea cucumber larvae at different developmental stage. Bars represent the relative percentage of each bacterial family. FE: Fertilized Egg; GL: Gastrula; EA: Early Auricularia; LA: Late Auricularia; PT: Pentactula; JN: Juvenile. **b** Family-level taxonomic distribution among rearing seawater of sea cucumber larvae at different developmental stage. FESW: Fertilized egg rearing seawater; GLSW: Gastrula rearing seawater; EASW: Early Auricularia rearing seawater; LASW: Late Auricularia rearing seawater; PTSW: Pentactula rearing seawater; JNSW: Juvenile rearing seawater

On family level, *Rhodobacteraceae* (22.9 ± 17.9%) and *Alteromonadaceae* (22.0 ± 19.6%) were the most dominant bacterial taxa during larval development, followed by *Nitrincolaceae* (7.7 ± 13.6%), *Flavobacteraceae* (6.2 ± 7.9%), *Marinobacteriaceae* (3.0 ± 3.5%) and *Methylophagaceae* (2.8 ± 6.2%). Before gut development, *Pseudoalteromonadacea* was significantly abundant with relative abundance of 9.9 ± 11.3% but rare in later stages with 0.2 ± 0.4%. Besides, *Colwelliaceae*, *Fusobacteriaceae*, *Saccharospirillaceae* and *Moraxellaceae* and *Idionarinaceae* were also prevalent in initial stages and decreased after gut development. In later stages, *Microbacteriaceae* was significantly increased from below detectable limit to 0.3 ± 0.3% before LA stage (Figs. 2c and 3). Furthermore, specific bacterial taxa at the family level that exhibited significant increases in abundance during specific developmental stages were observed (Figs. 2d and 3). *Pseudoalteromonadacea*, *Colwelliaceae* and *Shewanellaceae* were significantly abundant at FE stage; *Vibrionacaeae*, *Alcanivoracaceae* and *Saccharosorollaceae* were significantly abundant at the GL stage; *Rhodobacteraceae* and *Stappiaceae* significantly increased at the LA stage; *Bdellovibrionaceae* and *Nannocystaceae* significantly increased at the PT stage; *Hyphomonadaceae* and *Saprospiraceae* were significantly abundant at the JN stage.

### The impact of environmental microbiome on the dynamics of larval microbiome

**Fig. 4:**
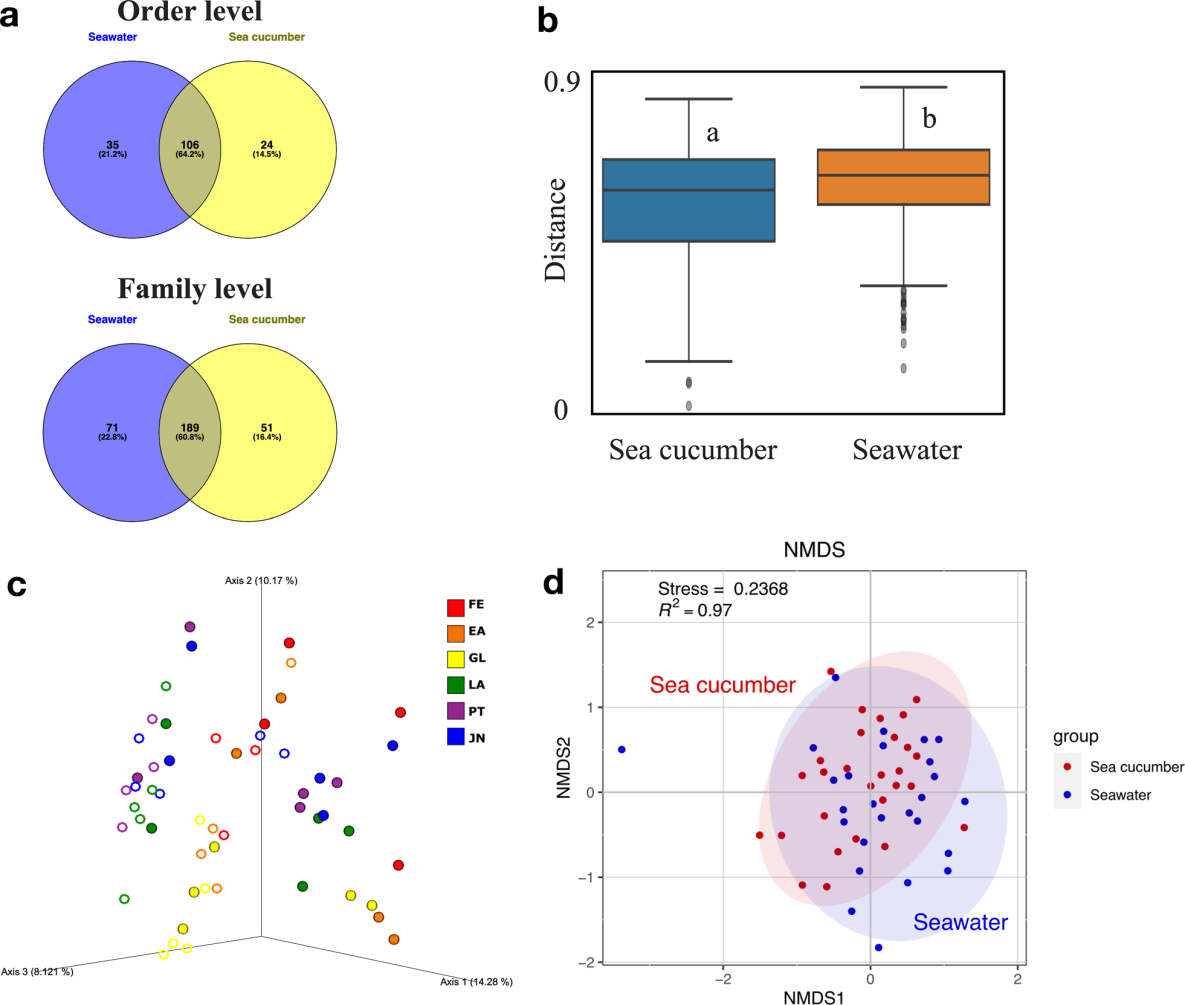
Microbiota shared within sea cucumber and seawater samples. **a** Venn diagram depicting unique and shared bacteria orders and families among sea cucumber larvae and their rearing seawater. **b** Boxplot based on unweighted UniFrac distance of atrophy larvae and their rearing seawater (PERMANOVA, *p* < 0.05, *q* < 0.05). Scale represents similarity within samples. **c** PCoA plot based on Unweighted UniFrac distance. Colors represent different developmental stages. Closed circles represent larvae samples and open circles represent seawater samples. **d** NMDS ordination based on Bray-Curtis distance showing the differences of microbiome between sea cucumber larvae and seawater

**Fig. 5:**
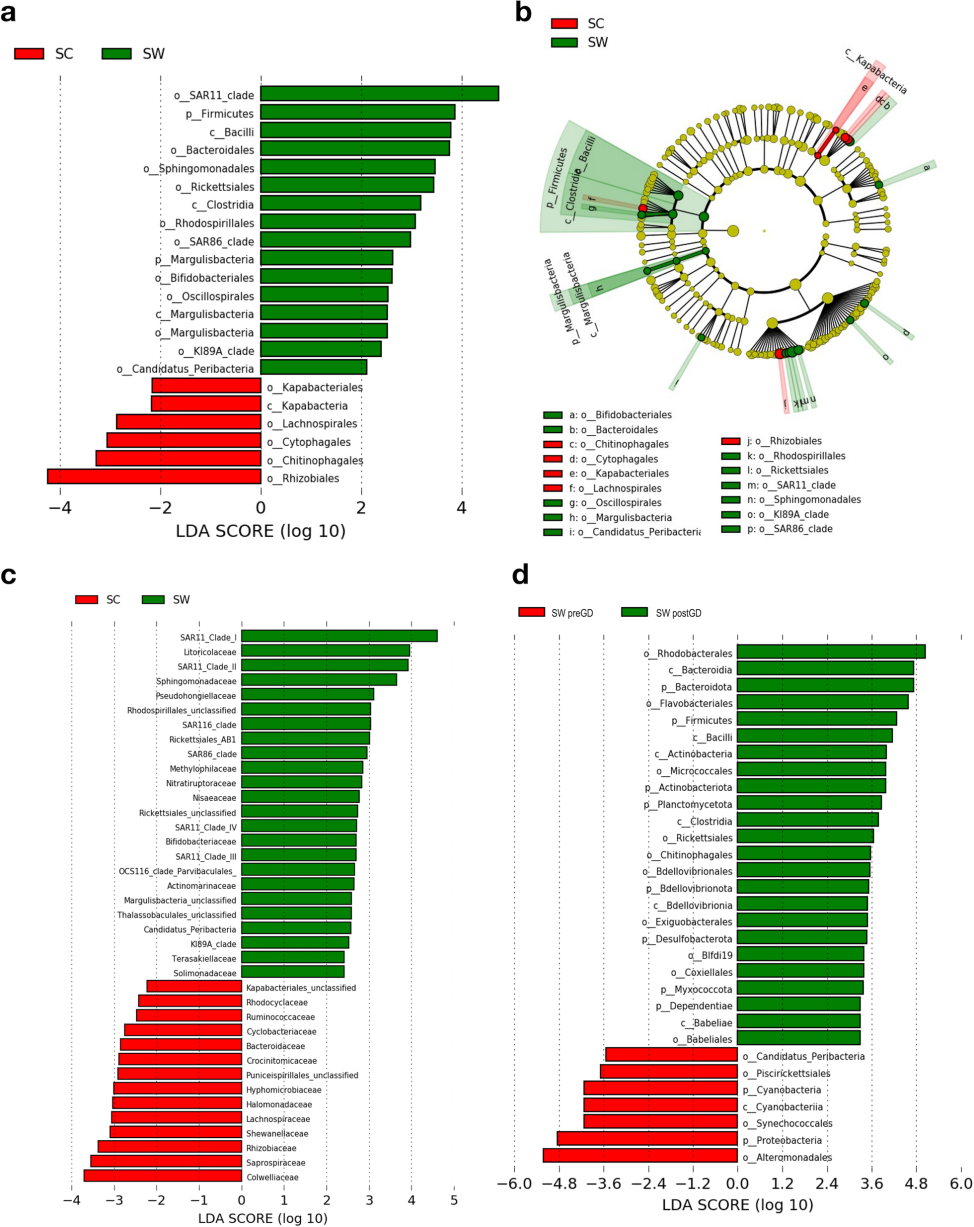
The linear discriminant analysis effect size (LEfSe) analysis of microbial abundance among sea cucumber larvae samples and their rearing seawater. **a** Taxa with significant differences in larvae and rearing seawater were detected by LEfSe analysis with a LDA threshold score of 3.5 and a p-value of 0.05. **b** The cladogram of detected prokaryotic taxa for microbial community of larvae and rearing seawater. **c** Only family level taxa with significant differences in larvae and rearing seawater detected by LEfSe analysis with a LDA threshold score of 3.5 and a p-value of 0.05. **d** Taxa with significant differences in rearing seawater pre- and post-gut development were detected by LEfSe analysis with a LDA threshold score of 3.5 and a p-value of 0.05

In order to understand if the rearing seawater is related to the dynamics of microbiome during larval development, the microbial structure and composition of rearing seawater were also assessed. Even though 64.2% bacterial taxa were shared between the rearing seawater and sea cucumber, the beta diversity based on unweighted and weighted UniFrac distance indicated the microbiota was significantly different between sea cucumber larvae and their rearing seawater (PERMANOVA, *p* < 0.01) (Fig. 4a–d and Additional file 2: Fig. S6). Particularly, *SAR11 clade* with relative abundance of 9.6 ± 20.0% was significantly dominant in rearing seawater but very rare in sea cucumber. *SAR86 clade* and *Rickettsiales* were also significantly abundant in rearing seawater with relative abundance of 0.2 ± 0.4% and 0.6 ± 0.9% but not prevalent in sea cucumber (Fig. 5a and b, and Additional file 1: Table S1). On a family level, 189 (60.8%) bacterial taxa were shared between rearing seawater and sea cucumber, however, 71 and 51 bacterial taxa were only detected in rearing seawater and sea cucumber, respectively. In which, some significant differences were also observed (Figs. 4a and 5c). Such as *SAR11 Clade I* (7.8 ± 16.0%), *Clade II* (1.6 ± 4.1%) and *Litoricolaceae* (1.5 ± 2.9%) were significantly abundant in rearing seawater but not detected or very rare in sea cucumber (Fig. 5c and Additional file 1: Table S2). The same tendency could also be observed in *Sphingomonadaceae, Pseudohongiellaceae, Methylophilaceae* and *Nitratiruptoraceae* (relative abundance in seawater > 0.1%).

Since the microbial composition of seawater is partly different from those to that of sea cucumber, we were speculating if the key bacteria taxa, which changed significantly along with the larval development, also changed in seawater. These bacteria taxa were evaluated to reveal whether the dynamics of microbiome in seawater is related to larval microbiome. Six bacterial taxa at order level in seawater were commensurate with the changes in sea cucumber larval microbiome, *Alteromonadales* (57.5 ± 34.9%) was significantly abundant before gut development and then decreased to 19.4 ± 18.5% in later stages, whereas *Rhodobacterales* (5.4 ± 8.0%), *Exiguobacterales* (0.2 ± 0.3%), *Micrococcales* (1.7 ± 1.2%), *Rickettsiales* (1.0 ± 1.0%) and *Blfdi19* (0.1 ± 0.1%) significantly increased in later stages (Figs. 2a and 5d). Moreover, SourceTracker analysis further unveiled the proportions of sources contributing to the composition of the larval microbiome (Additional file 2: Fig. S3). In both pre- and post-gut development stages, the influence of seawater accounted for 52% and 58% of the larval microbiome, respectively, whereas factors of unidentified origin affected 41% and 28% of the larval microbiome, respectively. These results underscore the impact of environmental seawater on the larval microbiome to a moderate extent, concurrently hinting at the involvement of other unidentified factors that drive alterations in the larval microbiome. According to the above findings, it becomes apparent that seawater alone might not solely determine the composition of the sea cucumber larval microbiome.

In addition, feeding is also considered as a possible factor that may affect microbiome of rearing seawater followed with the change larval microbiome, especially after gut development. At LA stage when feeding process started, bacteria taxa *Marivita*, *Kordia* and *Oceanicaulis* were significantly enriched in rearing seawater may influenced by input diets, which could explain the significant increase on the genus *Kordia* and *Marivita* at LA stage in sea cucumber larvae. However, other significantly increased bacteria in rearing seawater after diets induced were likely not related with the changes in larval microbiome at each developmental stage (Fig. 2e).

### Early life core microbiome and stage-core microbiome during larval development

**Fig. 6:**
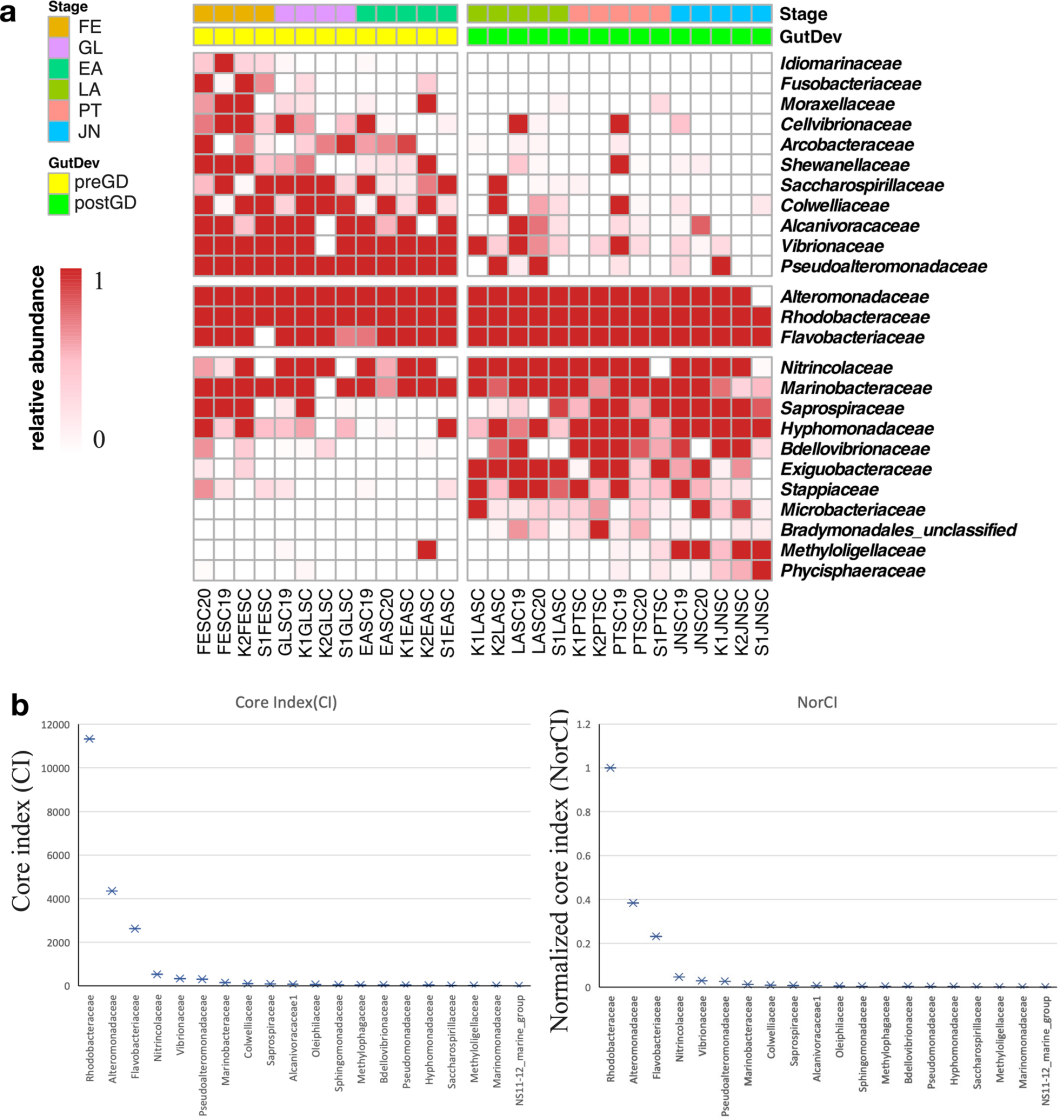
Detection of early life core microbiome and stage core microbiome. **a** Heatmap of core microbiome and stage core microbiome. Scale represents relative abundance. Scale represents relative abundance. Legend bar shows the sample developmental stage and gut development status. Stage shows the samples collected from different developmental stage. Dark yellow: fertilized egg, purple: gastrula, turquoise: early auricularia, dark green: late auricularia, pink: pentactula, and blue: juvenile, respectively. GutDev shows the gut development status of samples, light yellow represents samples before gut developed and light green represents samples post gut developed. FE: Fertilized Egg; GL: Gastrula; EA: Early Auricularia; LA: Late Auricularia; PT: Pentactula; JN: Juvenile. GutDev shows the gut development status of samples, light yellow represents samples before the gut developed and light green represents samples post-gut development. **b **CI for core bacteria (at the family level) in the sea cucumber larvae and NorCI, the normalized core index

Since the dynamics of microbial structure and significantly prevalent bacteria were encountered during larval development, we are wondering whether there is core microbiome shared during the early life of sea cucumber larvae. To define the core microbiome in our study, we employed a hybrid approach based on abundance and occupancy-based core microbiome analyses with core index (CI) calculation [32]. Specifically, we set up thresholds that collectively represented 75% of the total frequency (i.e., core taxa assigned to all taxa accounting for a substantial portion of the top 75% of total reads) and exhibited a presence in at least 70% of the total samples analyzed for abundance and occupancy-based analyses, respectively (Fig. 6a) [7]. For CI, we calculated CI in the top 20 most abundant families across all samples and the normalized index (NorCI) according to Zhang et al. [32]. This allowed us to extract more reliable core microbiome during the initial phases of larval development. Notably, families such as *Alteromonadaceae* and *Rhodobacteraceae*, exhibiting a > 0.3 of NorCI, were detected in over 93% of larval samples and collectively constituted 42.1% of the microbiome (Fig. 6b). These taxa were thereby identified as the early life core microbiome during the sea cucumber larval developmental stages.

Abundant taxa including *Vibrionaceae*, *Pseudoalteromonadaceae* and *Alcanivoracaceae* were also designated as the initial stage core microbiome (Additional file 2: Fig. S4). Conversely, *Nitrincolaceae* were recognized as later stage core microbiome constituents (Additional file 2: Fig. S4). Noteworthy bacterial families, such as *Pseudoalteromonadaceae*, *Colwelliaceae*, and *Shewanellaceae*, demonstrated significant enrichment at the FE stage through LEfSe analysis, while three additional families exclusively appeared at the FE stage, *Idiomarinaceae*, *Fusobacteriaceae*, and *Moraxellaceae*, and were subsequently subjected to CI analysis for pioneer core microbiome determination. Among these, *Pseudoalteromonadaceae* with > 0.3 NorCI were defined as pioneer core microbes (Figs. 2d and 6). Similarly, the JN stage exhibited significantly enriched bacterial families, *Hyphomonadaceae* and *Saprosiraceae*, along with two exclusive families, *Methyloligellaceae* and *Phycisphaeraceae*, all of which were selected for CI analysis (Figs. 2d and 6). In this context, *Methyloligellaceae* with > 0.3 of NorCI were designated as the juvenile core microbiome.

To gain deeper insights into the core microbiome contributing to host physiology, we conducted a more detailed analysis at both the genus and amplicon sequence variant (ASV) levels. Utilizing abundance and occupancy-based analyses, we identified the genera *Sulfitobacter, Marinobacter, Kordia*, and *Alteromonas* as core members at ASV levels (Additional file 1: Tables S3 and S4, Additional file 2: Fig. S2). The NorCI further validated ASV level core microbiome definition. Core microbiome ASV0004, ASV0005, ASV0007, and AS0010 were detected in fertilized eggs, gastrula and early auricularia, which were animal stages in pre-gut-development before start-feeding with a diatom (Additional file 2: Fig. S2).

### Meta-pangenomics of growth promoting bacteria *Sulfitobacter* BL28

**Fig. 7:**
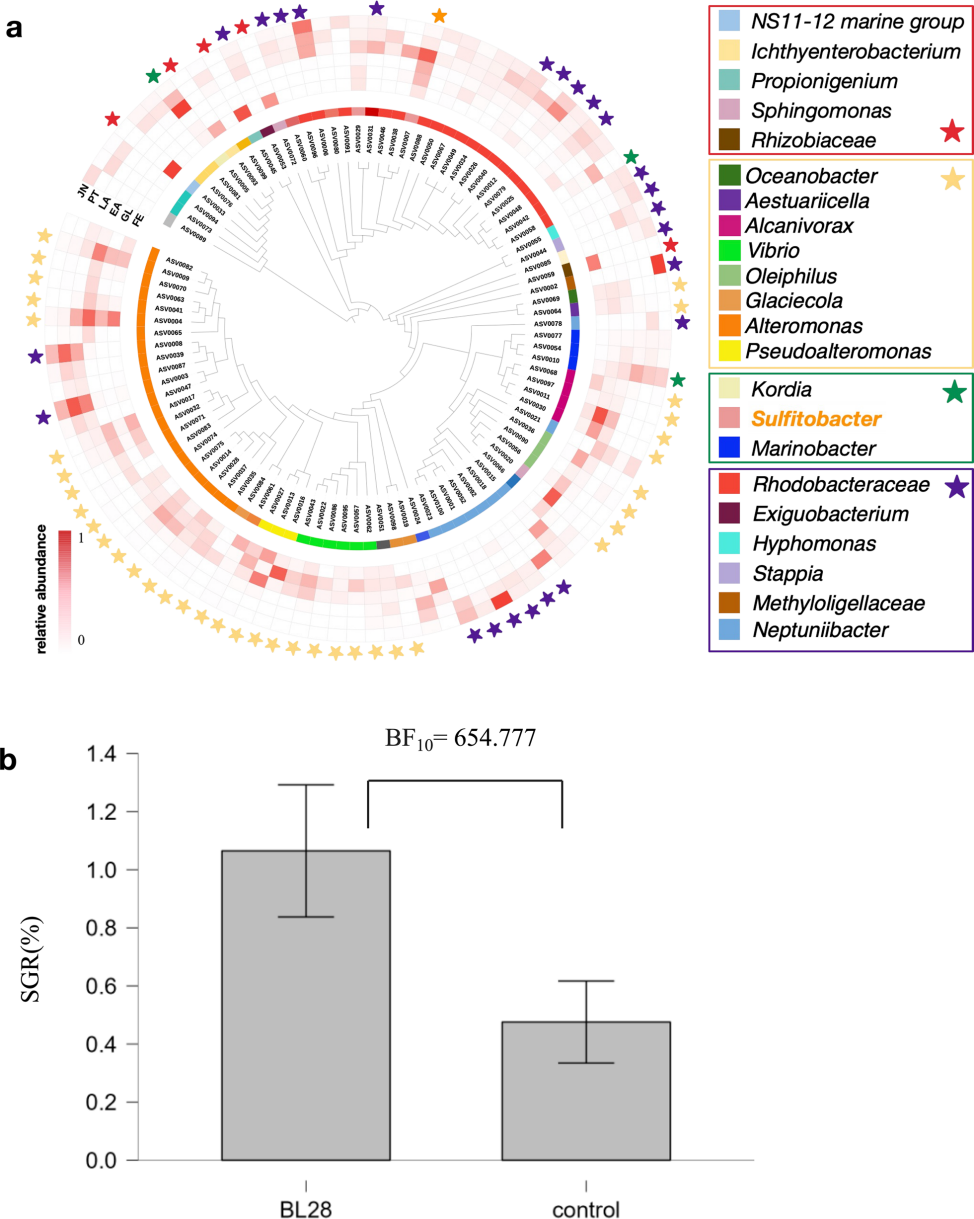
Growth promoting ability of *Sulfitobacter* sp. BL28. **a** A phylogenetic tree based on ASVs having > 500 reads with relative abundance. Inner circles represent taxonomic analysis at genus level. Red colored heatmap represent relative abundance of ASVs in sea cucumber samples. FE: Fertilized egg; GL: Gastrula; EA: Early auricularia; LA: Late auricularia; PT: Pentactula; JN: Juvenile. Star represents the key feature at different developmental stage. Red: fertilized egg; yellow: stages before gut developed; green: late auricularia; purple: stages post gut development; orange: *Sulfitobacter*. **b **Growth performance of juvenile sea cucumber with diet supplementary BL28. Bayes *t*-test with independent samples was performed using the JASP version 0.17.0 and growth with BL28 > those of control was set as an alternative hypothesis (H1), respectively. Alternative hypothesis was more likely to be occurred by 655 folds, respectively (*n* = 15). The error bar indicates standard error

Given that the results revealed the presence of universal core microbiome associated with larval developmental stage, it was of interest to investigate whether there were specific bacteria playing a vital role in the host biology during the early life stage. To enhance our understanding, we used bacterial isolates with sequences matched to key ASVs isolated from the 2019 rearing batch for testing host sea cucumber growth promotion [26] (Fig. 7a and Additional file 2: Fig. S2). Remarkably, laboratory experimental feeding tests revealed that BL28 showed apparent growth promotion of host sea cucumber, which was strongly supported based on Bayes factors (BF_10_ = 654.777) (Fig. 7b). The complete genome sequencing revealed that *Sulfitobacter* strain BL28 possesses the PHB metabolism gene cluster (Additional file 2: Fig. S1).

**Fig. 8:**
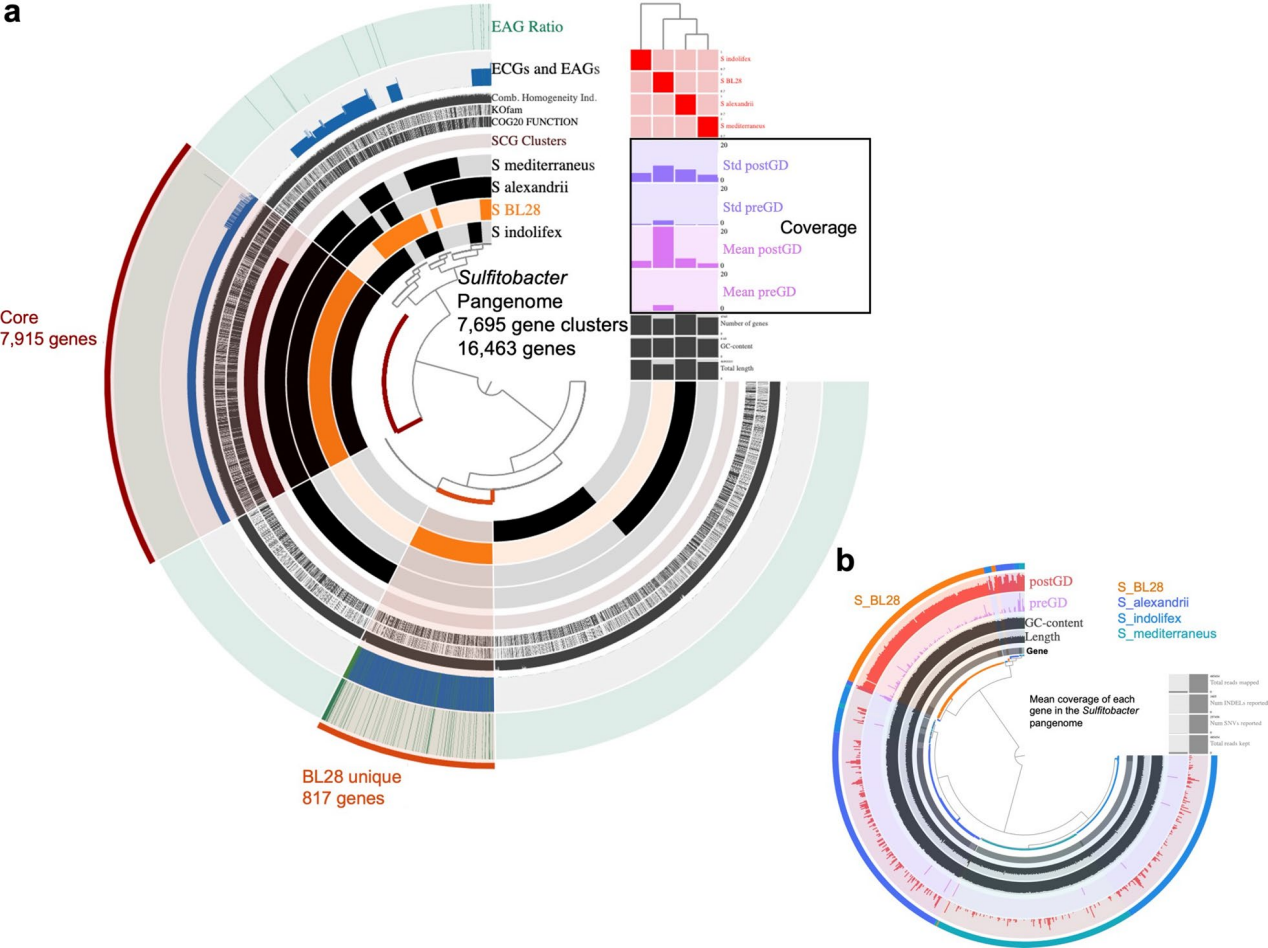
Meta-pangenomic analysis of *Sulfitobacter *sp. reveals its coverage in microbiota before and post gut development. **a** Pangenomics of *Sulfitobacter* sp. indicated the unique and core genes in BL28, and the higher total genome coverage in the post gut development. The inner radial dendrogram shows the gene clusters in the pangenome, clustered by presence/absence across genomes. The four genomes of *Sulfitobacter* strains are plotted on the innermost four layers, spaced to reflect discernable groups based on genomic composition. The genome pointed in orange is BL28 isolated from sea cucumber microbiome. Gene clusters within a given genome are filled in with black or dark orange; gene clusters do not present remain unfilled or light orange. Core gene across the *Sulfitobacter* strains were pointed in red and unique gene detected in BL28 were pointed in orange. Above the genome content summaries, each genome’s median coverage across larval metagenomes with different gut development status is shown in the colored bar graph. ANI value of genome is shown in the heatmap above, where each row represents a different sample, and cell color intensity reflects the ANI value. The colored two layers show the proportion of genes within each gene cluster determined to be environmental accessory or core genes: EAGs (green) and ECGs (blue) with 23 metagenomes. **b **Mean coverage of each gene in the pangenome of *Sulfitobacter *sp. within two developments indicated higher coverage in metagenomes of post-gut development environment. Outer layers show the four genomes of *Sulfitobacter *spp.and the coverage in environmental metagenomes. PGD, post gut development; BGD, before gut development

To further investigate the role of *Sulfitobacter* during the larval development stage, the complete genome of BL28 and 23 metagenomes obtained in this study, as well as three reference genomes collected from NCBI, were used for meta-pangenomic analyses (Fig. 8). Firstly, a pangenome using four genomes of genus *Sulfitobacter* was constructed, which consists of 7, 695 gene clusters with 16,463 genes, in which 7, 915 genes were recognized as core genes of *Sulfitobacter* pangenome and 817 genes were identified as unique genes of BL28 (Fig. 8A). The functional annotation for unique genes in BL28 showed that these unique genes were related to functions of amino acid biosynthesis, fatty acid biosynthesis, carbohydrate transport and metabolism, and cobalamin/B12 biosynthesis. Then, the pangenome was linked into the 23 environmental metagenomes, which were divided into two groups, pre-gut development (preGD) and post-gut development (postGD), by checking each gene’s median coverage across the metagenomes. Results showed that the total coverage of each *Sulfitobacter* genome in postGD environments (average 7.7) was significantly higher than preGD environments (average 0.8), in particular, the highest coverage was identified in the genome of BL28 in postGD (around 20). Meanwhile, the core genes of pangenome and unique genes of BL28 were identified as environmental core genes (ECGs) since they were covered in both preGD and postGD environments, which means the origination of BL28 environmental and further on/in hosts. Furthermore, each gene of not only BL28 but also the other three genomes of *Sulfitobacter* showed higher coverage in postGD compared to preGD environments, which may explain the abundant taxa in the later stage (Fig. 8B). The present findings suggest that *Sulfitobacter* BL28 may have a specific function in the early stages of larval development and host growth in sea cucumbers than the others. Additionally, BL28 belongs to *Rhodobacterales* may be particularly influential in promoting host growth from the initial stages in sea cucumber.

### Functional profile of early life core microbiome and stage-core microbiome

**Fig. 9:**
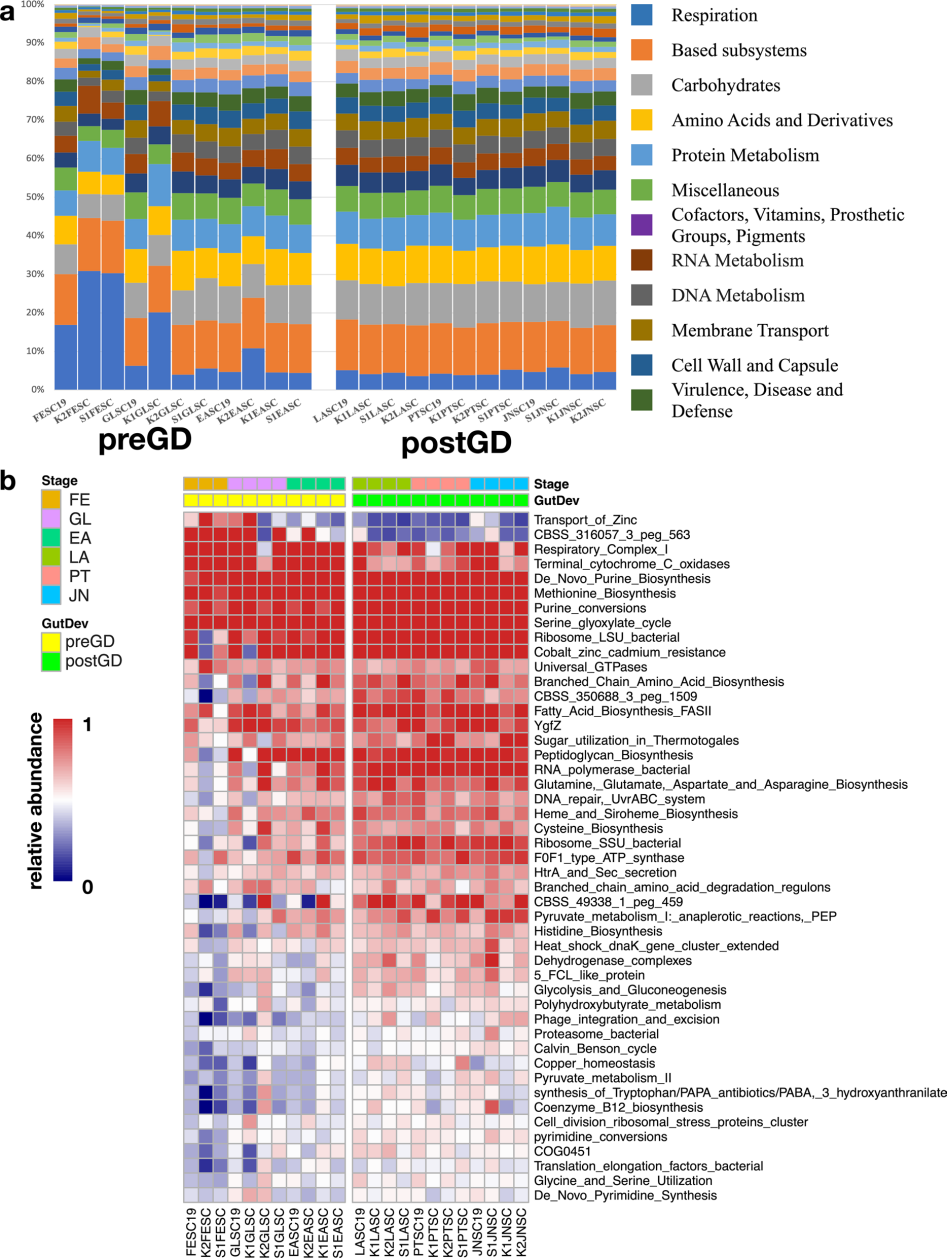
Microbial function analysis of larvae among different developmental stages. **a** Distribution of microbial functions among larvae samples before and post gut developed. Bars represent the relative percentage of each microbial functions. **b** Dynamics of significantly changed microbial functions pre- and post-gut development. Scale represents relative abundance. Legend bar shows the sample developmental stage and gut development status. Stage shows the samples collected from different developmental stages. Dark yellow: fertilized egg, purple: gastrula, turquoise: early auricularia, dark green: late auricularia, pink: pentactula, and blue: juvenile, respectively. GutDev shows the gut development status of samples, light yellow represents samples before gut developed and light green represents samples post gut developed

To better understand the role of the microbiome and determine how the functional capacity of the larval microbiome develops during early life stages, microbial functions at different developmental stages were analyzed. The top 10 core microbial functions are respiration, based subsystems, carbohydrates, amino acids and derivatives, protein metabolism, miscellaneous, cofactors, vitamins, prosthetic groups, pigments, RNA metabolism, DNA metabolism and membrane transport, in which the functions related to respiration is relatively higher at the FE stage (Fig. 9a and b). Whereas after gut development, feeding behavior and nutrient intake enriched functions belong to amino acid metabolisms, such as Glutamine and Asparagine biosynthesis, Cysteine biosynthesis, Histidine Biosynthesis and Glycine and Serine utilization, significantly increase in later stages. Pyruvate metabolism, sugar utilization and Glycolysis and Gluconeogenesis related to fats, proteins and carbohydrates metabolisms also increase in later stages after the feeding process. In addition, some metabolisms which are essential for carbon and energy intake, such as Polyhydroxybutyrate metabolism and Peptidoglycan biosynthesis, are enriched after gut development. The functional capacity of larval microbiome also varies from the developmental stage and developed with the organogenesis and feeding behavior during microbiome establishment.

## Discussion

Core microbiome plays a crucial role in maintaining normal host development and host health and facilitating adaptations to its environment [11–14]. However, ambiguous definition of core microbiome remains an obstacle to discovering beneficial microbes to host organisms, in particular, in marine invertebrates which share evolutionary history with humans and contribute to fisheries and aquaculture industries. Our study adopted a sophisticated abundance and occupancy-based core microbiome mining approach with 75% representation of total reads abundance and > 70% occupation thresholds [7] and further supported by core index [32]. Moreover, we expanded our fertilized egg sample diversity obtained from different years and sites, aiming to find a robust core microbiome unaffected by temporal and spatial variations, which is a less-explored aspect in previous core microbiome research. This approach successfully revealed ASV0007, which is affiliated to the host growth-promoting *S. pontiacus* strain BL28 isolated from *A. japonicus* blastula, as one of the cores of early life sea cucumber *A. japonicus*. Furthermore, our pioneering use of meta-pangenomics examined specific bacteria-host associations in the early life microbiome of 28 sea cucumber larval samples, shedding light on their metabolic roles in marine invertebrates. The study also highlighted the power of meta-pangenomics in understanding bacterial contributions to host physiology during early life development.

Sea cucumber (Echinodermata, Holothuroidea) is a member of the Deuterostomia clade, which also includes humans and other mammals. The shared ancestry of sea cucumbers and humans in the early deuterostome lineage underscores the significance of comprehending the evolutionary history and origin of Deuterostomia. The dynamics of gut microbiome in early life infants, mice and other marine organisms has been investigated intensively in recent years and is mainly shaped by host genetics, feeding mode, early environmental exposure, and birth mode, which has profound effects on the immune system, resistance to pathogens and absorption of nutrients [33–35]. Our study revealed the dynamics of larval microbiome during early colonization in sea cucumber related to host development, feeding behavior and environmental factors: (1) there is a significant change occurring at the late auricularia stage when the gut developed and feeding started; (2) bacteria that significantly increased at specific stages were detected; (3) microbiome of environmental rearing seawater is significantly different to larval microbiome; (4) temporal dynamics of key ASV using the growth-promoting *Sulfitobacter* strain BL28 genome. Studies in newborns revealed a large alteration in gut microbiota occurred during the transition in the diet from milk to solid food, in which the dominant bacteria *Bifidobacteria* and *Lactobacillus* shift to *Bacteroides* and *Firmicutes* [1]. The change in juvenile diets also altered the gut microbiome especially reducing the relative abundance of *Muribaculum intestinale* in mice [3]. Other studies of larval microbiome during early life stages of lake sturgeon and catfish indicate dynamic relationships between the gut microbiota composition and host ontogenesis such as gastrointestinal physiology [36, 37]. According to these studies, the significantly changed microbiome at the late auricularia stage after feeding is probably related to diets, development of gastrointestinal (GI) tracts and maturation of microbial functions for nutrition intake. Interestingly, we also demonstrated significantly increased bacteria at specific developmental stages indicating not only feeding behavior impacts on the larval microbiome, but also ontogenesis and metamorphosis shifts in the bacterial community.

Research in microbial colonization in infant revealed the establishment of microbiome during early life are essential to immune development and neurodevelopment and is related with diseases including IBD (Inflammatory Bowel Disease), obesity and asthma [2, 38]. As the pioneer microbiome is considered a crucial period for host biological development, extensive research has been dedicated to studying its composition, colonization mechanisms, and the various factors influencing this process [39]. Studies of Amphibian larvae have demonstrated that microbial colonization at hatching is essential for composition and function of the microbiome, which similar to the effects of birth mode on the human microbiome [40]. *Pseudoalteromonas* has been reported as potentially promoting larval settlement and metamorphosis in marine invertebrates through production of inductive cues consisting of molecular domains of polysaccharides, proteins, and lipids [41]. *Pseudoalteromonas* (*Pseudoalteromonadacea*) was extracted as a member of the pioneer core of sea cucumber microbiome. These significantly enriched pioneering bacteria on fertilized eggs are probably related to the metamorphosis of fertilized eggs in later developmental stages. Three members belonging to initial stage core microbiome *Vibrionaceae* were also regarded as core microbiome in early larval stages of Pacific blue shrimp, indicating these bacteria may play an important role in early life development and establishment of microbiotas [42]. The stage core microbiome during the sea cucumber larval development provides new insights into interactions of host microbiome and its physiology including ontogenesis and metamorphosis.

Core microbiomes are the key microbes shared across host populations and contribute to their host function and fitness, which is one of the best ways to understand the microbial composition and host-microbe interactions in early life stages [6–8]. In this study, we demonstrated early life core microbiome and stage-core microbiome during larval ontogenesis, enabling us to advance our understanding of the stability and dynamics of early life microbiome in sea cucumber and the further approaches of probiotics could benefit aspects of sea cucumber aquaculture such as pathogen resistance and microbiome mediation. Other studies on core microbiome in sea cucumber also indicated the change of core bacteria affiliated to hosts’ ecological and physiological events. A study revealed the seasonal core microbiome whose abundance are temporally stable across the host population during the events, in which *Proteobacteria* and *Bacteroidetes* increased in autumn and *Firmicutes* dominant in spring [13]. Studies of sea cucumber gut regeneration reported the temporary core microbiome at each generation stage [24, 25, 43]. In which, Yamazaki et al. [24] revealed the core microbiome during gut regeneration process include *Colwelliaceae*, *Flavobacteraceae*, *Rhodobacteraceae*, *Alteromonadaceae* and *Oceanospirillaceae*. Our studies and other studies in sea cucumber *A. japonicus* emphasized *Rhodobacteraceae*, a member of early life core microbiome, significantly increased after gut development during larval ontogenesis. *Rhodobacteraceae* has been widely studied for its potential positive effects on host immunity, growth, and pathogen defense [23, 44, 45]. In experiments of feeding behavior on growth rate of *A. japonicus*, *Rhodobacteraceae* are significantly abundant in fast-growing individuals related to capacity of algal polysaccharide degradation [46]. *Paracoccus* belonging to *Rhodobacteraceae* could promote sea cucumber intestinal microbiota homeostasis through improving modularity, enhancing species-species interactions and increasing the number of connecters within the network [47]. The study of dynamics of gut microbiota during gut regeneration by Yamazaki et al. pointed out *Sulfitobacter* belonging to *Rhodobacteraceae* significantly changed, highlighting the importance of *Sulfitobacter* in intestinal function recovery [23].

*Sulfitobacter* related genomes were dramatically increased after gut development in sea cucumber early life microbiome in our meta-pangenomic analyses, which is probably related to functional development such as nutrient intake that is also observed in infants. Lactic acid metabolisms were prevalent in infants during breast feeding but developing to an adult-like microbiota with higher nutrient availability when solid food was introduced [5]. The microbial functions of sugar utilization, fatty acid metabolism, amino acid metabolism, PHB metabolism and peptidoglycan biosynthesis are significantly enriched in later developmental stages. *Sulfitobacter* as a PHB producer, the increased function of PHB metabolism might coincide with the relative abundant *Sulfitobacter* in later developmental stages [48]. Besides, *Sulfitobacter* could be used as probiotics to inhibit the growth of fish pathogen *Vibrio anguillarum* [49]. The sea cucumber under antibiotics exposure showed a significantly changed microbiota with decreased abundance of *Sulfitobacter*, increasing the risk of infection [50]. The results of our study suggest that *S. pontiacus* BL28 has potential as a probiotic for improving the seed production of *A. japonicus* and contributing to its aquaculture. Further investigation of its probiotic ability to promote host growth and resistance against pathogens may lead to more effective application in aquaculture practices.

This study also firstly applied meta-pangenomics that combined both metagenomic and pangenomic methods to study the specific bacteria in early life microbiome in marine invertebrates. The unique genes of *S. pontiacus* BL28 were affiliated to functions of higher availability of nutrient intake and metabolism of essential vitamins, playing an important role in host oncogenesis and health maintenance. Meta-pangenomics studies have advanced our understanding of microbial populations and their functions. For example, research on *Prochlorococcus* revealed core gene clusters related to sugar metabolism, offering insights into their ecological roles [27]. Investigations into the oral microbiome demonstrated that different oral habitats are associated with specific functions, such as oxaloacetate decarboxylase metabolism in a tongue-enriched subgroup of *H. parainfluenzae* [28]. In humans, meta-pangenomics of *Escherichia* in older Chinese individuals highlighted higher abundance in the gut microbiome, with implications for dietary habits and gut health during aging [29]. The meta-pangenomics give us new insights into bacteria genome in its host and/or environmental metagenomes and its microbial functions related to host physiology and habitat preferences. Our meta-pangenomics of *Sulfitobacter* BL28 is just a beginning in the approach to understanding the role of specific bacteria in larval development [51], further exploration of other stage denoted microbes applied with meta-pangenomics could contribute to a more comprehensive and deep-seated knowledge of early life microbiome during sea cucumber ontogenesis.

## Conclusion

Our study applied metagenomic analysis on the early life microbiome using replicated larvae samples from different years and host populations, revealed the dynamics and stability of early life microbiome during sea cucumber larval development. Gut development and feeding behavior are the major factors impacting the alteration of early life microbial composition and functions occurring at the late auricularia stage, linked to the higher availability of nutrient digestive in gut during larval development. The early life core microbiome and stage core microbiome, including pioneer core microbes on fertilized egg, initial and later stage core microbiome, detected in larval microbiome might play an important role in larval ontogenesis and microbial homeostasis in sea cucumber, providing candidates to investigate potential probiotics contributing to improving sea cucumber seed production and aquaculture. Further studies using omics-approach of holobionts and visualization of core microbes on host and in environments are promoted to a deeper understanding of the interaction between bacteria and the host sea cucumber.

## Methods

### Sample collection and rearing conditions under laboratory conditions

Fertilized egg or gastrula samples of the sea cucumber *A. japonicus* were collected five times in 2019, 2020 and 2021. Fertilized eggs or gastrula were prepared at 18.7 °C at a farm of Hokkaido Aquaculture Promotion Corporation Kumaishi Branch, located in Kumaishi, Hokkaido, Japan (Latitude: 42.12574, Longitude: 139.99966) at 11:00 am on 5th August 2019, 4th August 2020, 30th June 2021 and 12th July 2021. Another fertilized egg sample was prepared at 18.7 °C at a farm in Hokkaido Aquaculture Promotion Corporation Shiriuchi Branch, located in Shiriuchi, Hokkaido, Japan (Latitude: 41.61463, Longitude: 140.38335) at 11:00 am on 30th July 2021. These eggs or gastrulas were transferred to the Laboratory of Microbiology, Faculty of Fisheries Sciences, Hokkaido University, whilst being kept at 18 °C for 2 h, and then used immediately for experiments. Density of the fertilized eggs or gastrula was set at 7500 eggs/L in an 8 L volume aquarium after manual counting of these eggs or gastrulas in 0.1 mL seawater using a microscope (AxioImager Z2, Zeiss, Oberkochen, Germany) and reared at 18 °C. The aquarium was prepared using a sterilized 8 L glass bottle (Ishizuka glass Co. Ltd., Aichi, Japan) set in an incubator (MLR-352-PJ, PHC Corp., Tokyo, Japan). In 2019, each rearing bottle was filled with 7.5 L natural filtrated seawater using a 50 μm mesh cartridge filter (SWP50P10, AS One, Osaka, Tokyo) used in the Kumaishi farm. In 2020 and 2021, each rearing bottle was filled with 7.5 L artificial sterilized seawater (SeaLife, Nihonkaisui Co., Ltd., Tokyo). Each 80 mL (7500 individuals) of fertilized egg or gastrula suspensions, corresponding to the final density of around 1 individual/mL, was added to each bottle, and rearing was started with aeration (SPP-25GA, Techno Takatsuki Co., Ltd., Osaka, Japan). Feeding started at 48 h after fertilization when early auricularia morphogenesis was observed in over 80% of individuals. A commercially available diatom, *Chaetoceros gracilis* (Hakodate Fisheries Research, Japan), was fed to the sea cucumber larvae daily. The diatoms used for feeding were alive and suspended in rearing seawater, allowing the free-swimming larvae to consume them naturally. We provided 8000 live diatom cells per individual larva per day. The rearing seawater for individual groups was not changed throughout the larval development stages.

### Subsampling of sea cucumber larvae and microbes in rearing water

Sea cucumber larvae at five major developmental stages (GL, EA, LA, PT and JN), were used for characterization of microbiomes after being confirmed by microscopic observation. Five liters of rearing seawater containing sea cucumber larvae were passed through a sterilized 40 μm nylon mesh (Falcon Cell Strainer, Durham, USA) to selectively isolate the larvae while effectively excluding diatoms, which are approximately 5 μm in size. The isolated larvae were subsequently rinsed once with 0.22 μm filter-sterilized seawater to ensure purity. Sterivex filter (SterivexTM-GV Sterile Vented Filter Unit 0.22 μm, EMD Millipore, Billerica, USA) was used to prepare this sterilized seawater. The larvae on nylon filters were immediately frozen at − 80 °C until DNA extraction.

To prepare microbial fractions in rearing water, five liters of rearing water after passing through nylon mesh was filtered through a 0.22 μm Sterivex filter by positive pressure using filtered (0.22 μm) N_2_ gas. These Sterivex filters were preserved at − 80 °C until DNA extraction.

### Microbial DNA extraction and 16 S rRNA gene sequencing

Microbial DNA extraction from sea cucumber was performed using the NucleoSpin Soil Kit (MACHEREY-NAGEL, Düren, Germany), according to the manufacturer’s protocol. Microbial DNA extraction from seawater was performed using the NucleoSpin Tissue kit (MACHEREY-NAGEL), according to the modified manufacturer’s protocol. In brief, seawater samples were heated at 55 °C for 1 h to add an active cell lysis process in TE buffer (10 mM Tris-HCl, 1 mM EDTA) containing 20% SDS and proteinase K (20 mg/mL) instead of buffer T1. In the third step Lyse Sample, 1 mL buffer B3 was used instead of 200 µL amount of the buffer.

The bacterial community was specifically targeted by amplifying the hypervariable V1-V2 region of the 16 S rRNA gene and accomplished using PCR with barcoded 27Fmod and 338R primers, which were also affixed with Illumina adaptor sequences [23]. Thermal cycling consisted of initial denaturation at 96 °C for 2 min, followed by 25 cycles of denaturation at 96 °C for 30 s, annealing at 55 °C for 45 s and extension at 72 °C for 1 min, and final extension at 72 °C on a 9700 PCR system (Life Technologies Japan, Tokyo, Japan). PCR amplicons were purified using AMPure XP magnetic purification beads (Beckman Coulter, Brea, CA, USA), and quantified using the Quant-iT PicoGreen dsDNA Assay Kit (Life Technologies Japan). Equal amount of each PCR amplicon was mixed and then sequenced using MiSeq Reagent Kit v3 (600-cycles) with the MiSeq Illumina platform. Based on sample specific barcodes, obtained reads were assigned to each sample.

### 16 S rRNA gene analysis and core microbiome analysis

The paired-end sequence data with quality scores (i.e., Fastq files) was analyzed using Quantitative Insights Into Microbial Ecology 2 (QIIME 2, version 2022.2) [52]. Quality controls (e.g., trimming primers and denoising sequences, removing chimeric sequences) and merging paired-end sequences were performed using DADA2 [53]. Reads with 100% similarity constituted an amplicon sequence variance (ASV). Unlike the method to cluster sequences into operational taxonomic units (OTUs) with fixed threshold (usually 97%), this quality control method using DADA2 allows us to detect even a single nucleotide difference. Taxonomic assignments for the ASVs were carried out using a Naive Bayes classifier trained on the Greengenes database. To enhance the specificity of our microbial community analyses, we systematically identified and excluded sequences corresponding to mitochondrial and chloroplast. For downstream diversity analyses, we subsampled reads to a minimum depth of 5683 per sample, which was enough number diversity saturated (Additional file 2: Fig. S5).

To dissect the complex interplay of microbial communities, multiple analytical methods were applied. Unweighted UniFrac distances served as beta-diversity metrics and were visualized using Principal Coordinate Analysis (PCoA) plots [54]. In addition, Bray–Curtis distance metrics were calculated to assess microbial community dissimilarities [55], and visualized through Non-metric Multidimensional Scaling (NMDS) using the ‘vegan’ and ‘ggplot2’ R packages [56, 57]. The potential influence of rearing seawater on the larval microbiome was assessed using the SourceTracker package [58].

Permutational Multivariate Analysis of Variance (PERMANOVA) was conducted to statistically validate the differences in UniFrac distances, with significance set at an FDR-corrected *p*-value of less than 0.05. Phylogenetic relationships were explored using FastTree [59]. Z-scores were calculated using the ‘genefilter’ R package [60]. Both ‘Phyloseq` and ‘DESeq2’ were employed for heatmap construction and subsequent statistical analyses [61, 62].

For identifying taxonomic differences, we used Linear Discriminant Analysis Effect Size (LEfSe), setting the *p*-value at 0.05 and LDA score at 3.5, per established guidelines [63]. To visualize shared Amplicon Sequence Variants (ASVs) between sea cucumber and seawater samples, Venn diagrams were generated using Venny 2.1 [64].

Given the absence of a consensus definition for the core microbiome, our study aligned with the latest findings [6, 7]. We opted for a hybrid definition, incorporating both occupancy and abundance-based criteria. In our analysis, core microbiome members were identified if they were present in over 70% of the total samples and accounted for a substantial portion, specifically 75%, of the total reads.

For a more refined understanding, we incorporated the Core Index (CI) and Normalized Core Index (NorCI) metrics, as introduced by Zhang et al. [32]. The CI is calculated using the formula:

$$Core Index\left(CI\right)=\frac{f*n*s}{N*S}$$

Where *f* represents the frequency of each family within the dataset, *n* is the number of samples containing each family, *s* is the sequence length for each family across all samples, *N* is the total number of samples, and *S* is the total sequence length from all samples.

The NorCI is then computed as:

$$NorCI=\frac{CI^{\prime }-{CI}_{min}}{{CI}_{max}-{CI}_{min}}$$

Here, *CI’* is the specific CI for each taxon, while *CI*_*min*_ and *CI*_*max*_ are the minimum and maximum CI values within the dataset, respectively.

For the purpose of this study, taxa with a NorCI greater than 0.3 were deemed to be part of the core microbiome in each taxon. This approach offers a robust and nuanced framework for defining the core microbiome, combining both ubiquity and relative abundance across samples.

### Metagenomic shotgun sequencing and functional profile

A total of 23 templates with ≥ 300 ng DNA material extracted from sea cucumber larvae collected in 2019 and 2021 were used for paired-end shotgun metagenomic sequencing on the HiSeq platform. For analysis of sea cucumber larval metagenome samples, the metagenomics RAST server (MG-RAST) was used for microbial functional annotation [65]. The low-quality regions were trimmed using SolexaQA then followed with dereplication by k-mer approach [66]. Duplicate Read Inferred Sequencing Error Estimation (DRISEE) was used to analyze the sets of Artificial Duplicate Reads (ADRs) [67, 68]. Reads with 97% identity were clustered and the longest sequence was picked as the cluster representative. The cluster representative was further assigned to taxonomy using a BLAT similarity search which integrates SILVA, Greengenes and RDP [69–71]. After the abundance profile generated, the SEED subsystem was used for functional profile [72].

### Probiotic experiment with *Sulfitobacter* BL28

Bacterial strain BL28 was isolated from sea cucumber larvae at the blastula stage and subsequently identified as *S. pontiacus* [26]. This strain was cultured on Marine Agar media (DifcoTM Marine Broth, Becton Dickinson, Franklin Lake, USA) at 20 °C. Commercial feed was obtained from Hokkaido Aquaculture Promotion Corporation, Japan. The experimental diet was prepared by adding 0.05 g of powder feeds to 1 mL of artificial seawater and supplementing it with a suspension of BL28 at a final concentration of 10^4^ CFU/mL. To prepare the suspension of BL28, the strain was incubated in Marine broth for 24 h at 20 °C, followed by centrifugation at 7000×*g* for 10 min at 15 °C. The resulting cell pellets were washed three times with sterile artificial seawater, and the suspension of BL28 was adjusted to a concentration of 1 × 10^8^ CFU/mL in artificial seawater.

Juvenile sea cucumbers were obtained from a farm of Hokkaido Aquaculture Promotion Corporation Kumaishi Branch, Japan, and acclimated to rearing conditions for one week. Thirty sea cucumbers were randomly distributed into six aquaria. The water temperature was maintained at 18 °C, salinity at 3.3–3.5%, and pH at 7.8–8.0. During the 30-day experiment, feeding was performed twice a week, and the tank was cleaned twice a week before feeding. The body length of the sea cucumber was measured before and after the experimental period to evaluate changes in body length. The sea cucumber was anesthetized using a 40% menthol solution (8 mL of 100% menthol and 12 mL of sterilized artificial seawater) to measure its body length. The specific growth rate (SGR) was calculated for statistical analysis using the following formula: SGR (%)=100×((Ln *L*_*t*_ − Ln *L*_0_)/t, where *L*_*t*_ and *L*_0_ are the final and initial length of sea cucumbers, respectively, and *t* is the experimental period of 30 days. The obtained body length data were analyzed using Bayes statistics to test whether null hypothesis (H0), which is the effect size = 0, was more probably occurred or not than the alternative hypothesis (H1) based on bayes factor (BF_10_) using JASP software version 0.17.0 [73].

### Meta-pangenomics workflow against ***Sulfitobacter*** isolates

Three currently existing complete genomes of genus *Sulfitobacter* from RefSeq database, National Center for Biotechnology Information (NCBI) and one assembled genome of *S. pontiacus* strain BL28 from this study were used for pan-genomic analysis, and then further combined with the above 23 metagenomes (divided into two groups: pre and post) for meta-pangenomic analysis. These analyses were mainly constructed using anvi’o v7.1 following methods from Delmont TO and Eren AM [27] and Utter et al. [28] with minor modifications. Briefly, the pan-genomic analysis of the four genomes was performed as previously described [74]. The genomes were first converted into an anvi’o-compatible database (anvi-gen-contigs-db). Further HMM decoration (anvi-run-hmms), COG annotations(anvi-run-ncbi-cogs) and KEGG annotation (anvi-run-kegg-kofams) for contig databases were performed. The pangenome was constructed through generating a genome storage (anvi-gen-genomes-storage) and followed with pangenome computing (anvi-pan-genome). For the metagenomes, firstly, the config files for illumina-utils were created (iu-gen-configs) and filtered with default (iu-filter-quality-minoche). Next, these metagenomes were mapped against the *Sulfitobacter* genomes to create the bam files using bowtie2-2.3.5 [75], sorted and indexed using samtools-1.16.1 [76], profiled using anvi-profile, and merged to an anvi’o profile database (anvi-merge). Then, gene coverage values across metagenomes per genome were calculated (anvi-summarize). Finally, the meta-pangenomics were generated by linking the pangenome to the metagenomes (anvi-meta-pan-genome). The results were visualized (anvi-display-pan) and improved manually.

### Supplementary Information

**Additional file 1: Table S1.** Relative abundance of each bacteria taxon at order level. SC: Sea cucumber; SW: Seawater; preGD: before gut development; postGD: post gut development. **Table S2.** Relative abundance of each bacteria taxon at family level. SC: Sea cucumber; SW: Seawater; preGD: before gut development; postGD: post gut development. Orange represents the number represent samples' occupancy >70%; yellow represents samples' abundance in top 75% of total reads. **Table S3.** Relative abundance of each bacteria taxon at genera level in sea cucumber samples. preGD: before gut development; postGD: post gut development. Orange represents the number represent samples' occupancy >70%; yellow represents samples' abundance in top 75% of total reads. **Table S4.** Relative abundance of ASVs in sea cucumber samples. preGD: before gut development; postGD: post gut development. Orange represents the number represent samples' occupancy >70%; yellow represents samples' abundance in top 75% of total reads.

**Additional file 2: Fig. S1.** Genome analysis revealed the pathway of PHB metabolism.**Fig. S2.** Heatmap based on relative abundance of top100 ASVs.**Fig. S3.** The impact of seawater on microbiome of sea cucumber larvae analyzed by SourceTracker. **a** Barplot of the proportion of sources in specific sample. **b** Pie plot of proportion of sources in larval microbiome before and after gut development. SW-Before: seawater before larval gut development; SW-Post: seawater post larval gut development.**Fig. S4.** Normalized core index (NorCI) for core bacteria in the sea cucumber larvae. **a** NorCI of bacteria at family level in sea cucumber larvae before gut development. **b** NorCI of bacteria at family level in sea cucumber larvae post gut development. **c** NorCI of bacteria at genera level in sea cucumber samples. **d** NorCI of ASVs in sea cucumber samples. **Fig. S5.** Rarefaction curve of **a** sea cucumber samples and **b** seawater samples.**Fig. S6.** Beta diversity of weighted UniFrac distance of larval microbiome. **a** PCoA plot based on weighted UniFrac distance showing the microbiota differences in different years. Different color represent different years; closed circle represent larvae samples before gut development, triangle represent larvae samples post gut development. **b** Weighted UniFrac distance plot between different developmental stage. **c** Weighted UniFrac distance plots between different years and **d** between sea cucumber before and post gut development (PERMANOVA, *p*<0.05). **e** PCoA plot based on weighted UniFrac distance of seawater and sea cucumber microbiotas. Different color represent developmental stages; closed circle represent larval samples; open circles represent the seawater samples. **f** Weighted UniFrac distance plot between sea cucumber and seawater (PERMANOVA, *p*<0.05, **q**<0.05).

## Data Availability

Sequences were deposited to DDBJ/ENA/GenBank under accession numbers as DRA012782 and DRA015867-DRA015868 for meta16S and metagenome, and AP027373-AP027377 for BL28 complete genome.

## Abbreviations

ASVs: Amplicon sequence variant
LEfSe: LDA effect size
LDA: Liner discriminant analysis
PCoA: Principal coordinate analysis
BUL: below undetectable limit
FE: Fertilized egg
GL: Gastrula
EA: Early auricularia
LA: Late auricularia
PT: Pentactula
JN: Juvenile
SC: Sea cucumber
SW: Seawater
preGD: Pre gut development
postGD: Post gut development

## References

[1] Kapourchali FR, Cresci GAM (2020). Early-life gut microbiome—the importance of maternal and infant factors in its establishment. Nutr Clin Pract.

[2] Arrieta MC, Stiemsma LT, Amenyogbe N, Brown E, Finlay B (2014). The intestinal microbiome in early life: health and disease. Front Immunol.

[3] McNamara MP, Singleton JM, Cadney MD, Ruegger PM, Borneman J, Garland T (2021). Early-life effects of juvenile Western diet and exercise on adult gut microbiome composition in mice. J Exp Biol.

[4] Sun Z, Lee-Sarwar K, Kelly RS, Lasky-Su JA, Litonjua AA, Weiss ST, et al. Identifying the critical time window for the association of the early-life gut microbiome and metabolome with childhood neurodevelopment. medRxiv. 2022.

[5] Bäckhed F, Roswall J, Peng Y, Feng Q, Jia H, Kovatcheva-Datchary P (2015). Dynamics and stabilization of the human gut microbiome during the first year of life. Cell Host Microbe.

[6] Neu AT, Allen EE, Roy K (2021). Defining and quantifying the core microbiome: challenges and prospects. PNAS.

[7] Custer GF, Gans M, van Diepen LTA, Dini-Andreote F, Buerkle CA (2023). Comparative analysis of core microbiome assignments: implications for ecological synthesis. mSystems.

[8] Risely A (2020). Applying the core microbiome to understand host–microbe systems. J Anim Ecol.

[9] Wang Y, Gong J, Li J, Xin Y, Hao Z, Chen C (2020). Insights into bacterial diversity in compost: core microbiome and prevalence of potential pathogenic bacteria. Sci Total Environ.

[10] Zaura E, Keijser BJF, Huse SM, Crielaard W (2009). Defining the healthy core microbiome of oral microbial communities. BMC Microbiol.

[11] Wu S, Ou H, Liu T, Wang D, Zhao J. Structure and dynamics of microbiomes associated with the marine sponge *Tedania* sp. during its life cycle. FEMS Microbiol Ecol. 2018;94. 10.1093/femsec/fiy055 29617990

[12] van Cise AM, Wade PR, Goertz CEC, Burek-Huntington K, Parsons KM, Clauss T (2020). Skin microbiome of beluga whales: spatial, temporal, and health-related dynamics. Anim Microb.

[13] Feng J, Zhang L, Tang X, Xia X, Hu W, Zhou P (2021). Season and geography induced variation in sea cucumber (*Stichopus japonicus*) nutritional composition and gut microbiota. J Food Compos Anal.

[14] Leal Cv, Avelino-Alves D, Salazar V, Omachi C, Thompson C, Berlinck RGS (2022). Sponges present a core prokaryotic community stable across Tropical Western Atlantic. Sci Total Environ.

[15] Yang H, Hamel J-F, Mercier A (2015). The sea cucumber *Apostichopus japonicus*: history, biology and aquaculture.

[16] Oh G-W, Ko S-C, Lee DH, Heo S-J, Jung W-K (2017). Biological activities and biomedical potential of sea cucumber (*Stichopus japonicus*): a review. Fish Aquat Sci.

[17] Han Q, Keesing JK, Liu D (2016). A review of sea cucumber aquaculture, ranching, and stock enhancement in China. Rev Fish Sci Aquac.

[18] Chen C-Z, Li P, Liu L, Li Z-H (2022). Exploring the interactions between the gut microbiome and the shifting surrounding aquatic environment in fisheries and aquaculture: a review. Environ Res.

[19] Zhao Z, Jiang J, Zheng J, Pan Y, Dong Y, Chen Z (2022). Exploiting the gut microbiota to predict the origins and quality traits of cultured sea cucumbers. Environ Microbiol.

[20] Zhao Z, Jiang J, Pan Y, Dong Y, Wang B, Gao S (2022). Sea cucumber body vesicular syndrome is driven by the pond water microbiome via an altered gut microbiota. mSystems.

[21] Chi C, Liu J-Y, Fei S-Z, Zhang C, Chang Y-Q, Liu X-L (2014). Effect of intestinal autochthonous probiotics isolated from the gut of sea cucumber (*Apostichopus japonicus*) on immune response and growth of *A. japonicus*. Fish Shellfish Immunol.

[22] Zhao Y, Zhang W, Xu W, Mai K, Zhang Y, Liufu Z (2012). Effects of potential probiotic *Bacillus subtilis* T13 on growth, immunity and Disease resistance against *Vibrio splendidus* Infection in juvenile sea cucumber *Apostichopus japonicus*. Fish Shellfish Immunol.

[23] Yamazaki Y, Meirelles PM, Mino S, Suda W, Oshima K, Hattori M (2016). Individual *Apostichopus japonicus* fecal microbiome reveals a link with polyhydroxybutyrate producers in host growth gaps. Sci Rep.

[24] Yamazaki Y, Sakai Y, Yu JW, Mino S, Sawabe T (2020). Tracking the dynamics of individual gut microbiome of sea cucumber Apostichopus japonicus during gut regeneration. PeerJ.

[25] Yu Z, Xue Z, Liu C, Zhang A, Fu Q, Yang K (2021). Distinct microbiota assembly mechanisms revealed in different reconstruction stages during gut regeneration in the sea cucumber *Apostichopus japonicus*. MicrobiologyOpen.

[26] Yu JW, Sakai Y, Mino S, Sawabe T (2022). Characterization of microbiomes associated with the early life stages of sea cucumber *Apostichopus japonicus Selenka*. Front Mar Sci.

[27] Delmont TO, Eren AM (2018). Linking pangenomes and metagenomes: the *Prochlorococcus* metapangenome. PeerJ.

[28] Utter DR, Borisy GG, Eren AM, Cavanaugh CM, Mark Welch JL (2020). Metapangenomics of the oral microbiome provides insights into habitat adaptation and cultivar diversity. Genome Biol.

[29] Li J, Gui Q, Yang Y, He C, Huang S, Zhang X et al. Metapangenomics reveals an increased proportion of an *Escherichia coli*-dominated enterotype in older Chinese people. 2022. https://doi.org/10.21203/rs.3.rs-1344848/v1 10.1631/jzus.B2400341 PMC12119184 40436643

[30] Peña-Montenegro TD, Kleindienst S, Allen AE, Eren AM, McCrow JP, Sánchez-Calderón JD et al. *Colwellia* and *Marinobacter* metapangenomes reveal species-specific responses to oil and dispersant exposure in deepsea microbial communities. bioRxiv. 2020;317438.

[31] Boeuf D, Eppley JM, Mende DR, Malmstrom RR, Woyke T, DeLong EF (2021). Metapangenomics reveals depth-dependent shifts in metabolic potential for the ubiquitous marine bacterial SAR324 lineage. Microbiome.

[32] Zhang Q, Zhang Z, Lu T, Yu Y, Penuelas J, Zhu YG, Qian H (2021). Gammaproteobacteria, a core taxon in the guts of soil fauna, are potential responders to environmental concentrations of soil pollutants. Microbiome.

[33] Gschwendtner S, Kang H, Thiering E, Kublik S, Fösel B, Schulz H (2019). Early life determinants induce sustainable changes in the gut microbiome of six-year-old children. Sci Rep.

[34] Low A, Soh M, Miyake S, Seedorf H. Host-age prediction from fecal microbiome composition in laboratory mice. bioRxiv. 2020;412734.

[35] van Oppen MJH, Blackall LL (2019). Coral microbiome dynamics, functions and design in a changing world. Nat Rev Microbiol.

[36] Abdul Razak S, Griffin MJ, Mischke CC, Bosworth BG, Waldbieser GC, Wise DJ (2019). Biotic and abiotic factors influencing channel catfish egg and gut microbiome dynamics during early life stages. Aquaculture.

[37] Razak SA, Scribner KT (2020). Ecological and ontogenetic components of larval lake sturgeon gut microbiota assembly, successional dynamics, and ecological evaluation of Neutral community processes. Appl Environ Microbiol.

[38] Grier A, Qiu X, Bandyopadhyay S, Holden-Wiltse J, Kessler HA, Gill AL (2017). Impact of prematurity and nutrition on the developing gut microbiome and preterm infant growth. Microbiome.

[39] Perez-Muñoz ME, Arrieta MC, Ramer-Tait AE, Walter J (2017). A critical assessment of the sterile womb and in utero colonization hypotheses: implications for research on the pioneer infant microbiome. Microbiome..

[40] Warne RW, Kirschman L, Zeglin L (2017). Manipulation of gut microbiota reveals shifting community structure shaped by host developmental windows in Amphibian larvae. Integr Comp Biol.

[41] Peng LH, Liang X, Xu JK, Dobretsov S, Yang JL (2020). Monospecific biofilms of *Pseudoalteromonas* promote larval settlement and metamorphosis of *Mytilus coruscus*. Sci Rep.

[42] Giraud C, Callac N, Boulo V, Lam J-S, Pham D, Selmaoui-Folcher N et al. The active microbiota of the eggs and the nauplii of the Pacific blue shrimp *Litopenaeus stylirostris* partially shaped by a potential vertical transmission. Front Microbiol. 2022;13;886752. 10.3389/fmicb.2022.886752 PMC9133551 35633721

[43] Weigel BL (2020). Sea cucumber intestinal regeneration reveals deterministic assembly of the gut microbiome. Appl Environ Microbiol.

[44] Grotkjær T, Bentzon-Tilia M, D’Alvise P, Dourala N, Nielsen KF, Gram L (2016). Isolation of TDA-producing *Phaeobacter* strains from sea bass larval rearing units and their probiotic effect against pathogenic *Vibrio* spp. in Artemia cultures. Syst Appl Microbiol.

[45] Henriksen NNSE, Lindqvist LL, Wibowo M, Sonnenschein EC, Bentzon-Tilia M, Gram L (2022). Role is in the eye of the beholder—the multiple functions of the antibacterial compound tropodithietic acid produced by marine *Rhodobacteraceae*. FEMS Microbiol Rev.

[46] Feng Q-M, Ru X-S, Zhang L-B, Zhang S-Y, Yang H-S (2022). Differences in feeding behavior and intestinal microbiota may relate to different growth rates of sea cucumbers (*Apostichopus japonicus*). Aquaculture.

[47] Yang G, Peng M, Tian X, Dong S (2017). Molecular ecological network analysis reveals the effects of probiotics and florfenicol on intestinal microbiota homeostasis: an example of sea cucumber. Sci Rep.

[48] Gnaim R, Polikovsky M, Unis R, Sheviryov J, Gozin M, Golberg A (2021). Marine bacteria associated with the green seaweed *Ulva* sp. for the production of polyhydroxyalkanoates. Bioresour Technol.

[49] Emilia Noor S, Eguchi M (2012). Mixed cultures of the phytoplankton *Nannochloropsis oculata* and the marine bacterium *Sulfitobacter* sp. RO3 inhibit the growth of virulent strains of the major fish pathogen *Vibrio anguillarum*. Aquac Sci.

[50] Zhao Y, Wang Q, Liu H, Li B, Zhang H (2019). High-throughput sequencing of 16S rRNA amplicons characterizes gut microbiota shift of juvenile sea cucumber *Apostichopus japonicus* feeding with three antibiotics. J Oceanol Limnol.

[51] Yamano R, Yu J, Jiang C, Harjuno Condro Haditomo A, Mino S, Sakai Y (2022). Taxonomic revision of the genus *Amphritea* supported by genomic and in silico chemotaxonomic analyses, and the proposal of *Aliamphritea* gen. Nov. PLoS One.

[52] Bolyen E, Rideout JR, Dillon MR, Bokulich NA, Abnet CC, Al-Ghalith GA (2019). Reproducible, interactive, scalable and extensible microbiome data science using QIIME 2. Nat Biotechnol.

[53] Callahan BJ, McMurdie PJ, Rosen MJ, Han AW, Johnson AJA, Holmes SP (2016). DADA2: high-resolution sample inference from Illumina amplicon data. Nat Methods.

[54] Lozupone C, Lladser ME, Knights D, Stombaugh J, Knight R (2011). UniFrac: an effective distance metric for microbial community comparison. ISME J.

[55] Beals EW (1984). Bray–Curtis ordination: an effective strategy for analysis of multivariate ecological data. Adv Ecol Res.

[56] Oksanen J, Kindt R, Legendre P, O’Hara B, Stevens MHH, Oksanen MJ (2007). The vegan package. Community Ecol Package.

[57] Villanueva RAM, Chen ZJ (2019). ggplot2: elegant graphics for data analysis.

[58] Knights D, Kuczynski J, Charlson ES, Zaneveld J, Mozer MC, Collman RG (2011). Bayesian community-wide culture-independent microbial source tracking. Nat Methods.

[59] Price MN, Dehal PS, Arkin AP (2010). FastTree 2—approximately maximum-likelihood trees for large alignments. PLoS One.

[60] Gentleman R, Carey V, Huber W, Hahne F. Genefilter: methods for filtering genes from high-throughput experiments. 2023. https://git.bioconductor.org/packages/genefilter.

[61] McMurdie PJ, Holmes S (2013). Phyloseq: an R Package for Reproducible Interactive Analysis and Graphics of Microbiome Census Data. PLoS ONE.

[62] Love MI, Huber W, Anders S (2014). Moderated estimation of Fold change and dispersion for RNA-seq data with DESeq2. Genome Biol.

[63] Segata N, Izard J, Waldron L, Gevers D, Miropolsky L, Garrett WS (2011). Metagenomic biomarker discovery and explanation. Genome Biol.

[64] Oliveros JC. Venny. An interactive tool for comparing lists with Venn’s diagrams. https://bioinfogp.cnb.csic.es/tools/venny/index.html.

[65] Meyer F, Paarmann D, D’Souza M, Olson R, Glass EM, Kubal M (2008). The metagenomics RAST server – a public resource for the automatic phylogenetic and functional analysis of metagenomes. BMC Bioinform.

[66] Cox MP, Peterson DA, Biggs PJ, SolexaQA (2010). At-a-glance quality assessment of Illumina second-generation sequencing data. BMC Bioinform.

[67] Keegan KP, Trimble WL, Wilkening J, Wilke A, Harrison T, D’Souza M (2012). A platform-independent method for detecting errors in metagenomic sequencing data: DRISEE. PLoS Comput Biol.

[68] Gomez-Alvarez V, Teal TK, Schmidt TM (2009). Systematic artifacts in metagenomes from complex microbial communities. ISME J.

[69] Cole JR, Chai B, Marsh TL, Farris RJ, Wang Q, Kulam SA (2003). The ribosomal database project (RDP-II): previewing a new autoaligner that allows regular updates and the new prokaryotic taxonomy. Nucleic Acids Res.

[70] DeSantis TZ, Hugenholtz P, Larsen N, Rojas M, Brodie EL, Keller K (2006). Greengenes, a chimera-checked 16S rRNA gene database and workbench compatible with ARB. Appl Environ Microbiol.

[71] Pruesse E, Quast C, Knittel K, Fuchs BM, Ludwig W, Peplies J (2007). SILVA: a comprehensive online resource for quality checked and aligned ribosomal RNA sequence data compatible with ARB. Nucleic Acids Res.

[72] Overbeek R, Begley T, Butler RM, Choudhuri Jv, Chuang H-Y, Cohoon M (2005). The subsystems approach to genome annotation and its use in the project to annotate 1000 genomes. Nucleic Acids Res.

[73] Love J, Selker R, Marsman M, Jamil T, Dropmann D, Verhagen J (2019). JASP: graphical statistical Software for Common Statistical designs. J Stat Softw.

[74] Jiang C, Kasai H, Mino S, Romalde JL, Sawabe T (2022). The pan-genome of *Splendidus* clade species in the family *Vibrionaceae*: insights into evolution, adaptation, and pathogenicity. Environ Microbiol.

[75] Langmead B, Salzberg SL (2012). Fast gapped-read alignment with Bowtie 2. Nat Methods.

[76] Danecek P, Bonfield JK, Liddle J, Marshall J, Ohan V, Pollard MO (2021). Twelve years of SAMtools and BCFtools. Gigascience.

