# Supplementary material for: Unveiling the early life core microbiome of the sea cucumber Apostichopus japonicus and the unexpected abundance of the growth-promoting Sulfitobacter
Source: Anim Microbiome. 2023 Oct 24;5:54. doi: 10.1186/s42523-023-00276-2 (PMC10599069; doi:10.1186/s42523-023-00276-2)
Supplement: Supplementary file 2 — Additional file 2: Fig. S1. Genome analysis revealed the pathway of PHB metabolism.Fig. S2. Heatmap based on relative abundance of top100 ASVs.Fig. S3. The impact of seawater on microbiome of sea cucumber larvae analyzed by SourceTracker. a Barplot of the proportion of sources in specific sample. b Pie plot of proportion of sources in larval microbiome before and after gut development. SW-Before: seawater before larval gut development; SW-Post: seawater post larval gut development.Fig. S4. Normalized core index (NorCI) for core bacteria in the sea cucumber larvae. a NorCI of bacteria at family level in sea cucumber larvae before gut development. b NorCI of bacteria at family level in sea cucumber larvae post gut development. c NorCI of bacteria at genera level in sea cucumber samples. d NorCI of ASVs in sea cucumber samples. Fig. S5. Rarefaction curve of a sea cucumber samples and b seawater samples.Fig. S6. Beta diversity of weighted UniFrac distance of larval microbiome. a PCoA plot based on weighted UniFrac distance showing the microbiota differences in different years. Different color represent different years; closed circle represent larvae samples before gut development, triangle represent larvae samples post gut development. b Weighted UniFrac distance plot between different developmental stage. c Weighted UniFrac distance plots between different years and d between sea cucumber before and post gut development (PERMANOVA, p<0.05). e PCoA plot based on weighted UniFrac distance of seawater and sea cucumber microbiotas. Different color represent developmental stages; closed circle represent larval samples; open circles represent the seawater samples. f Weighted UniFrac distance plot between sea cucumber and seawater (PERMANOVA, p<0.05, q<0.05). [file 42523_2023_276_MOESM2_ESM.pptx]

## Slide 1
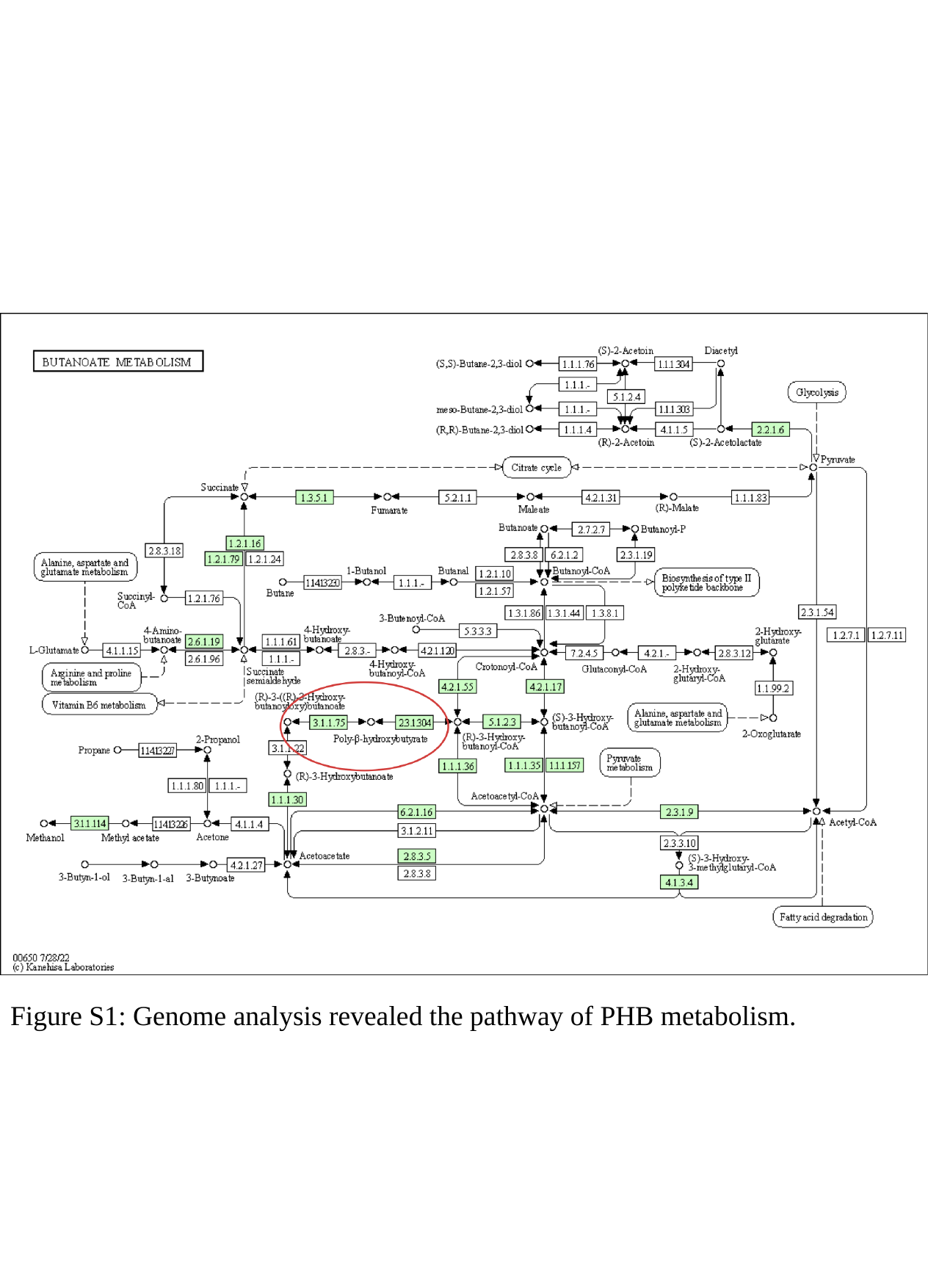

Figure S1: Genome analysis revealed the pathway of PHB metabolism.

## Slide 2
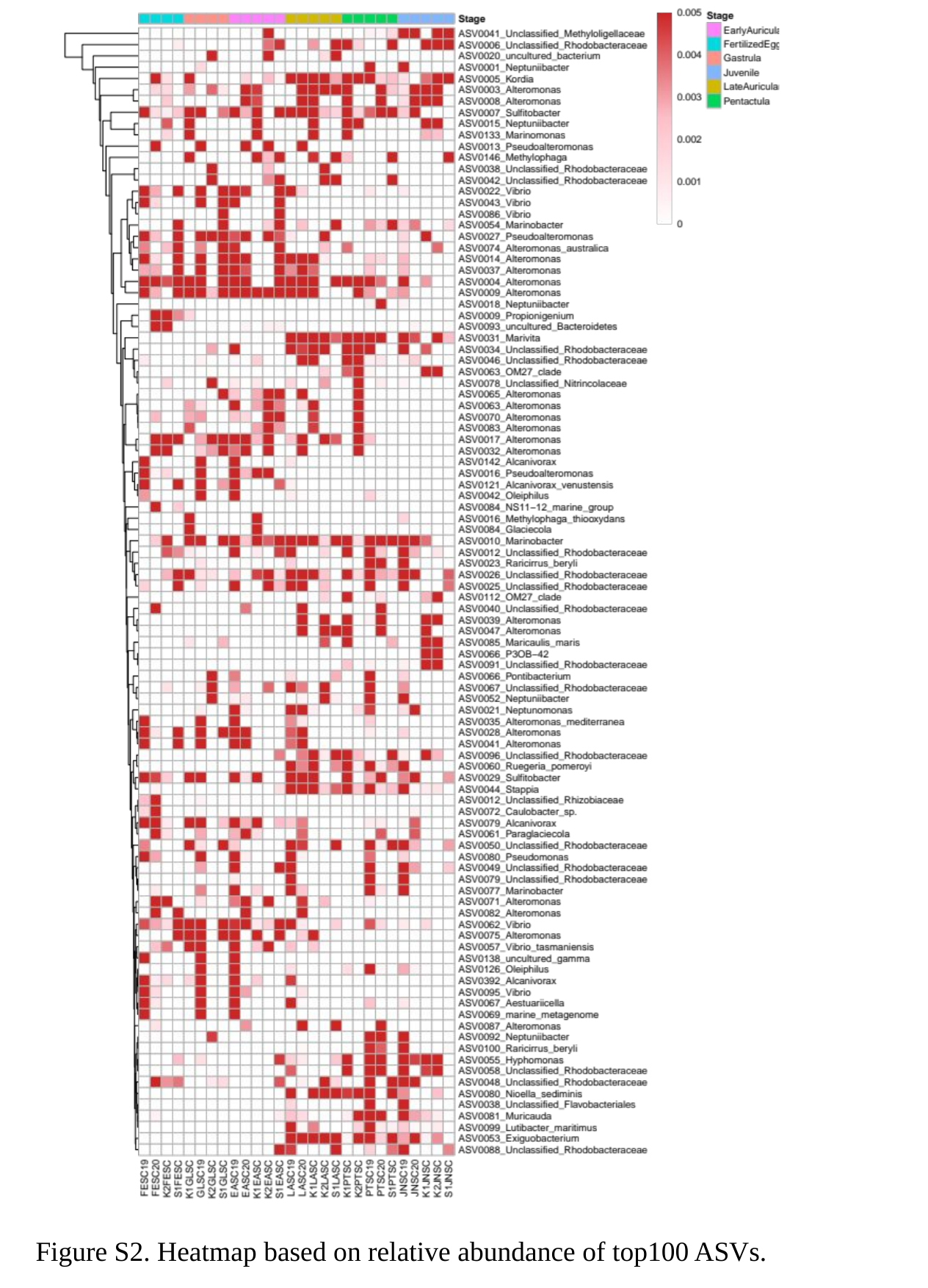

Figure S2. Heatmap based on relative abundance of top100 ASVs.

## Slide 3
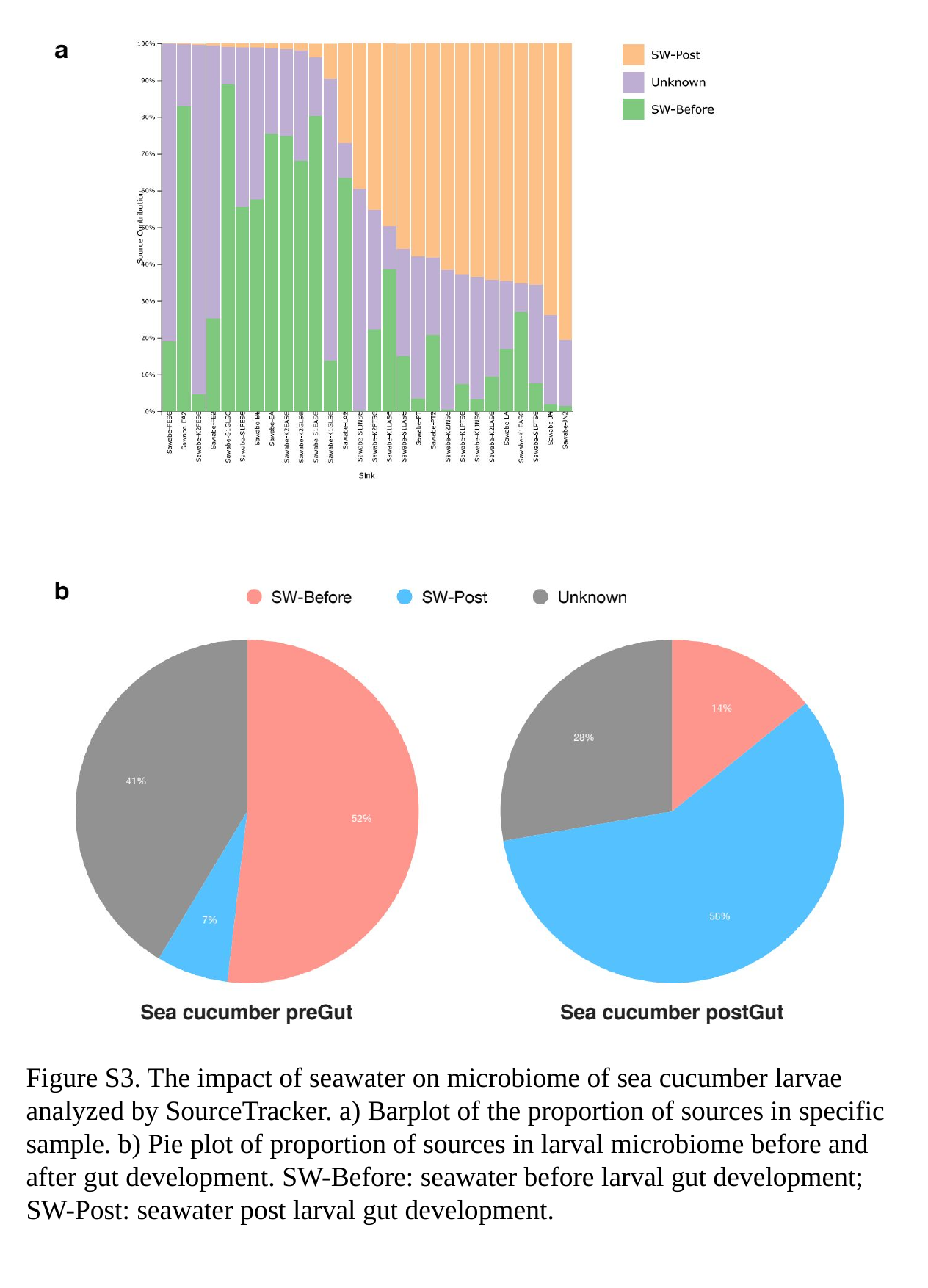

Figure S3. The impact of seawater on microbiome of sea cucumber larvae analyzed by SourceTracker. a) Barplot of the proportion of sources in specific sample. b) Pie plot of proportion of sources in larval microbiome before and after gut development. SW-Before: seawater before larval gut development; SW-Post: seawater post larval gut development.

## Slide 4
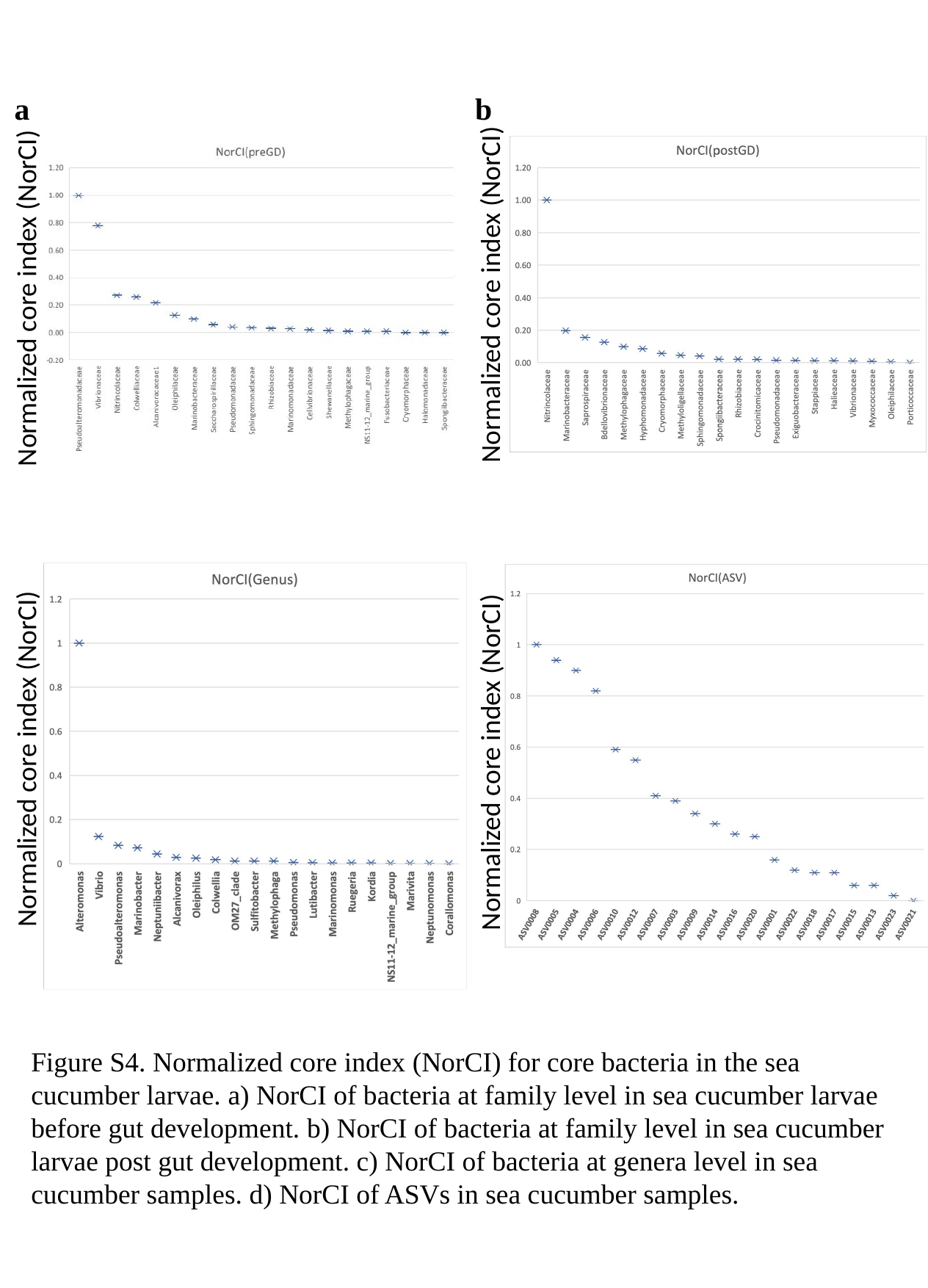

a
b
Normalized core index (NorCI)
Normalized core index (NorCI)
Normalized core index (NorCI)
Normalized core index (NorCI)
Figure S4. Normalized core index (NorCI) for core bacteria in the sea cucumber larvae. a) NorCI of bacteria at family level in sea cucumber larvae before gut development. b) NorCI of bacteria at family level in sea cucumber larvae post gut development. c) NorCI of bacteria at genera level in sea cucumber samples. d) NorCI of ASVs in sea cucumber samples.

## Slide 5
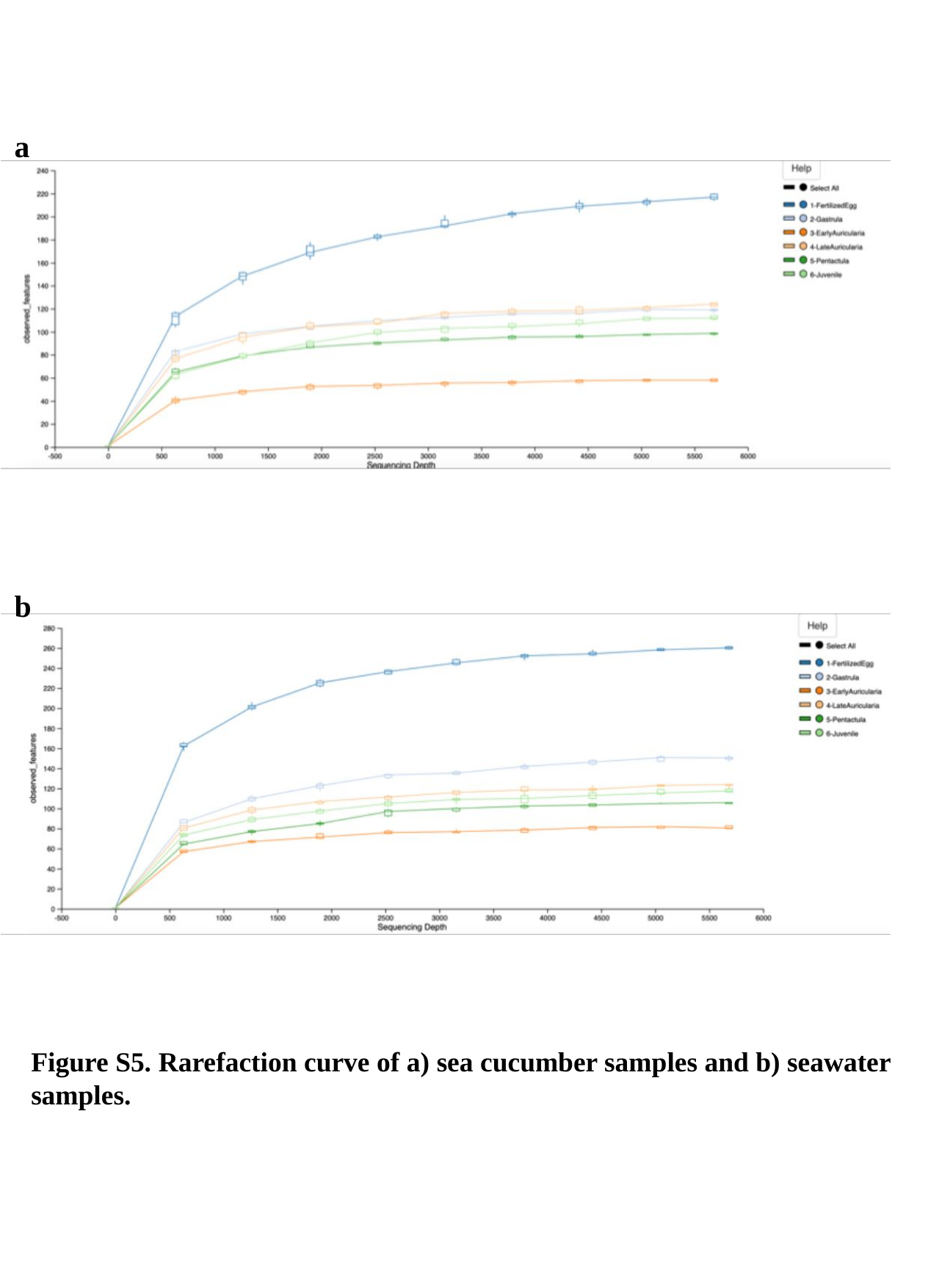

a
b
Figure S5. Rarefaction curve of a) sea cucumber samples and b) seawater samples.

## Slide 6
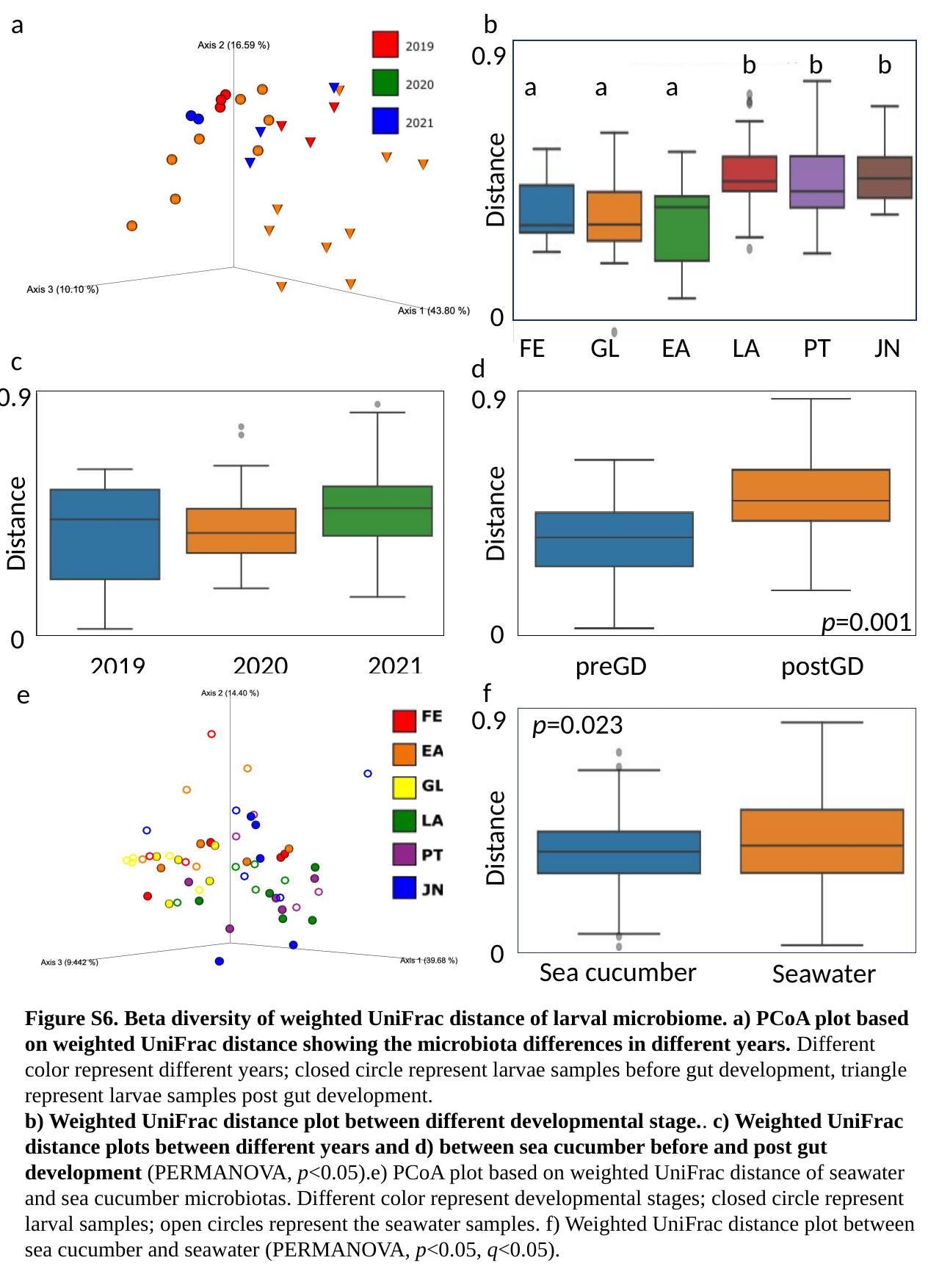

a
b
0.9
b
b
b
a
a
a
Distance
0
FE
GL
EA
LA
PT
JN
c
d
0.9
0.9
Distance
Distance
p=0.001
0
0
postGD
2020
2021
preGD
2019
f
e
0.9
p=0.023
Distance
0
Sea cucumber
Seawater
Figure S6. Beta diversity of weighted UniFrac distance of larval microbiome. a) PCoA plot based on weighted UniFrac distance showing the microbiota differences in different years. Different color represent different years; closed circle represent larvae samples before gut development, triangle represent larvae samples post gut development.
b) Weighted UniFrac distance plot between different developmental stage.. c) Weighted UniFrac distance plots between different years and d) between sea cucumber before and post gut development (PERMANOVA, p<0.05).e) PCoA plot based on weighted UniFrac distance of seawater and sea cucumber microbiotas. Different color represent developmental stages; closed circle represent larval samples; open circles represent the seawater samples. f) Weighted UniFrac distance plot between sea cucumber and seawater (PERMANOVA, p<0.05, q<0.05).
